# Multi-use of the sea: A wide array of opportunities from site-specific cases across Europe

**DOI:** 10.1371/journal.pone.0215010

**Published:** 2019-04-11

**Authors:** Martina Bocci, Stephen Joseph Sangiuliano, Alessandro Sarretta, Joseph Onwona Ansong, Bruce Buchanan, Andronikos Kafas, Mario Caña-Varona, Vincent Onyango, Eva Papaioannou, Emiliano Ramieri, Angela Schultz-Zehden, Maximilian Felix Schupp, Vassiliki Vassilopoulou, Marta Vergílio

**Affiliations:** 1 Thetis Spa, Castello, Venice, Italy; 2 Marine Scotland, Scottish Government, Marine Planning & Policy Division, Edinburgh, Scotland, United Kingdom; 3 CNR – National Research Council of Italy, ISMAR Institute of Marine Sciences / Arsenale, Castello, Venice, Italy; 4 s.Pro - sustainable projects, GmbH, Berlin, Germany; 5 Marine Scotland Science, Aberdeen, Scotland, United Kingdom; 6 University of the Azores, Ponta Delgada, The Azores, Portugal; 7 University of Dundee, Nethergate, Dundee, Scotland, United Kingdom; 8 Thetis Spa, Castello, Venice, Italy; 9 SUBMARINER Network for Blue Growth EEIG, Berlin, Germany; 10 Alfred Wegener Institute, Helmholtz Centre for Polar and Marine Research, Bremerhaven, Germany; 11 HCMR Hellenic Centre for Marine Research, Agios Kosmas Helliniko, Greece; 12 CIBIO – Research Centre in Biodiversity and Genetic Resources/InBIO – Associate Laboratory, University of the Azores, Ponta Delgada, The Azores, Portugal; University of Essex, UNITED KINGDOM

## Abstract

The concept of multi-use of the sea has gained popularity in recent years as a result of ocean space (coastal areas and regions with relatively small sea space in particular) becoming increasingly crowded due to the development of the maritime economy. Competing claims for space can be a source of conflict, however this may also lead to mutual benefits for different users when sustainable combinations are sought. Despite increasing European-wide efforts, on-the-ground knowledge and practice of multi-use are still limited. Therefore, with the aim of investigating opportunities for multi-use development in the European seas, 10 case studies were selected, involving different site-specific contexts. This study analyses the characteristics and development potential for ocean multi-use, integrating results from desk analysis and stakeholder perceptions from different sectors in each of the case study locations. Similarities and differences between various combinations of sea uses are also identified. The results show a high heterogeneity of multi-use opportunities between case studies, with a range of combinations identified. The investigated combinations of maritime uses share an overall balance between factors promoting (drivers) and hindering (barriers) multi-use development. Based on stakeholder opinions, expected benefits (added values) of multi-use implementation outweigh potential negative impacts. Management actions are also proposed to further exploit multi-use potential at a local, regional (sub-national) and national levels.

## Introduction

There is an ever increasing variety of commercial activities in European sea basins: oil and gas (O&G) extraction; renewable energy production; pipelines and cables; shipping; coastal and maritime tourism; fisheries; aquaculture and blue biotechnology, and sand and mineral extraction. Furthermore, European seas offer several intangible assets linked to the cultural and natural capital that they host, and the different services related to them (e.g. marine defence and oceanographic monitoring & research). To that end, under Blue Growth concepts and approaches [1], [2], maritime activities are expected to grow in the near future, which may intensify conflicts, particularly in cases where different uses compete for the same space.

In an effort to minimise the use of sea space, particularly in circumstances where space is limited, and to build synergies among sectors (e.g. sharing of costs and infrastructures, common licensing procedures), single uses should be concentrated wherever possible. This would make sea space available for other uses or would allow space to remain free from development. Limiting the use of sea space is an important element in marine planning. It is relevant in the here and now (e.g. to leave part of the marine ecosystem undisturbed, beyond its formal protection designation through various spatial-based protection measures), and also for the future, to deal with different aspects of a long-term planning perspective. Available sea space would help to respond to future, not yet fully known, spatial needs related to economic developments (e.g. international trade, transport and logistics, security of energy supply, research and innovation). It can also help deal with other future uncertain scenarios, such as those related to climate change, and the consequences of this in terms of resilience and adaptation of the socio-economic and environmental systems [3].

To address sustainable exploitation of ocean resources and use of sea space, the concept of Multi-Use (MU) was introduced. Some MU definitions are available in the literature. MU has been interpreted as co-location of complementary activities [4], or as the combination of different industries and technologies in ocean spaces [5]. The H2020 Multi-Use of European Seas (MUSES) project, of which this research is part of, has defined MU as an intentional joint use of resources in close geographic proximity (i.e. in the same area) [6].

The will to combine sea-uses has evolved from the possibility of MU to provide economic benefits to involved marine users (e.g. through sharing of costs for licensing, personnel, logistics, etc.), for example making an alternative source of revenue available for declining or restricted sectors, or enabling developments in maritime areas where this would otherwise not be possible (e.g. due to the dominance of other maritime uses). This can also lead to diversifying sectors which can provide additional socio-economic benefits to coastal communities [7].

From a marine spatial planning (MSP) perspective, MU can provide solutions to conflicts arising or increasing with the increasing exploitation of the oceans. These conflicts exist as both user-user conflicts, where competing sectors require use of the same space, and user-environment conflicts, where an activity negatively impacts the natural environment [8]. Environmental and exploitation conflicts are frequently about the access to, and use of, natural resources and the space as well as the distribution of the associated benefits and costs; this can involve actual or potential users. The harm that different co-located activities inflict upon each other through impacts on operation (e.g. offshore wind production and fisheries) or ecosystem impacts (e.g. tourism and environmental protection) can also be a source of conflict [9]. For this reason, creating solutions to conflicts represents a central issue in the context of ecosystem-based management, especially in densely used marine areas [9].

MU is also linked to the concepts of coexistence and synergistic use of the sea, both representing key issues in MSP. Coexistence is understood as the absence of conflict and the neutral sharing of marine space [10]. Synergy refers to mutually beneficial use of the same sea space or marine resources, but equally to shared infrastructure, technology or human resources [10]. Both concepts link to the issue of spatial efficiency (supporting more sustainable use of marine space) but also process efficiency to promote blue economy. A key driver for synergy between sectors is the expected efficiency gain and mutual benefit, so that the net benefit arising from the combination of uses is greater than the sum of their individual effects [10]. MU definitively represents an operational solution to promote synergies among sea uses.

However, there is still relatively little experience on how to integrate multiple uses and functions that share the same space at sea, and only limited knowledge is available on cumulative environmental effects on the marine environment. If it is well known how concentration of uses can determine cumulative effects on the marine environment (e.g. [11], [12], [13]), the way MU could reduce environmental impacts in specific scenarios needs to be assessed on a case-by-case basis. In fact, this can be achieved by developing environmentally sustainable MU combinations or by preserving some vulnerable areas from presence of human activities. For example, establishing a MU between tourism (e.g. diving) and environmental protection (Marine Protected Areas) can reduce the impacts due to non-regulated access to protected areas. Similarly, combining offshore renewable energy production with aquaculture can provide the opportunity to move offshore an activity (in this case aquaculture) potentially harming the quality of coastal waters. But comprehensive studies on this aspect are not yet available.

The concept of MU of the sea has already been discussed for almost two decades, e.g. combining offshore wind energy with aquaculture in the North Sea [14], [15], [16]. This combination was recently considered relevant for global food production, trade and electricity generation, as well as for its powerful social and ethical implications [17]. Several other examples of MU have also been studied in recent years, including: offshore wind energy production and environmental protection in coastal waters [18], [19]; offshore wind and wave energy production [20]; and fisheries and tourism (also referred as pescatourism) [21]. In 2010 the ‘The Ocean of Tomorrow’ cross-thematic calls were launched under the European Union (EU) 7th Research Framework Programme (FP7) with the intention of fostering multidisciplinary approaches on key cross-cutting marine and maritime challenges. Opportunities to explore MU platforms were created and exploited by three main projects: MERMAID (Innovative Multi-purpose offshore platforms: planning, design and operation [5], [22], [23], [24]); TROPOS (Modular Multi-use Deep Water Offshore Platform Harnessing and Servicing Mediterranean, Subtropical and Tropical Marine and Maritime Resources [25]), and; H2Oceans (Development of a wind-wave power open sea platform equipped for hydrogen generation with support for multiple users of energy [26]). These experiences—all foreseeing the realisation of engineering infrastructures at sea—evaluated in detail the technical and economic implication of feasibility.

Despite these research initiatives, a broader analysis of opportunities and barriers for the realisation of MU platforms and, more in general, for implementation of MU was still lacking. On the contrary, in order to advance commercial MU deployments, a clear understanding of challenges related to policy, permitting procedures, safety issues, investments, cross-sector dialogue and societal acceptance is crucial.

The need for a more clear understanding of potential for MU development is also linked to the differences in resources and capacity that can foster specific MU concepts across the European Sea Basins. Physical conditions, availability of space and ecological richness are important factors influencing the development of specific MU combinations. For example, MUs involving aquaculture, fisheries and environmental protection appear relevant across all sea basins, but MU with environmental protection are of particular significance in the Black and Baltic seas [7]. In southern Europe, MUs generally develops around tourism. Tourism-driven MUs usually involve co-location of uses where existing equipment or installation is used without major modifications (e.g. tourism and fishing). These exist already on a small-scale in coastal areas where tourism activities take place [7]. MUs that involve the energy sector and the use of fixed or floating offshore installations (e.g. offshore wind and aquaculture) are relevant largely in the northern part of Europe (north-eastern Atlantic, North Sea and the south-west Baltic Sea), given the advanced development of, and need for, offshore energy in these regions [7].

MU might not always be the best option for all sectors or areas of the sea, so it is fundamental to carefully consider local conditions when deciding whether single or multi-use should be favoured in a given location. Such a systematic assessment of MU on a local scale was also not available.

In order to fill the above gaps we assessed MU in 10 different case studies, exploring the variety of both already implemented and potential MU across different European locations. The objective is to identify which specific MU combinations have the greatest opportunity to be developed (or strengthened, if already in place) and which are the most beneficial in terms of environmental-socio-economic benefits at a local level. The study also aims to provide a more general understanding on what types of MU are considered as more promising across European seas and what maritime activities could drive MU development in local contexts. Finally, the study aims to identify key actions to be undertaken to practically promote MU development.

Given these objectives, the Research Issues (RIs) addressed through this study are:

**RI-1**. Identification of the most promising MU combinations to be strengthened (in case of existing ones) or developed (in case of new ones) in different local contexts throughout European Seas, given the local environmental and socio-economic characteristics.
**RI-2**. Identification of relevant technical, economic, environmental and societal factors for MU development (i.e. drivers and barriers to MU and expected added values and negative impacts of MU) and stakeholders driven evaluation of MU potential (based on the joint assessment of drivers and barriers) and MU overall expected effect (based on the joint assessment of added values and impacts).
**RI-3**. Identification of commonalities and contrasts (in terms of drivers, barriers, added values, impacts, MU potential and MU overall expected effect) amongst different combinations, groups of combinations and different local contexts.
**RI-4**. Stakeholder-driven identification of actions needed to support MU development, emerging from the site-specific contexts.

Site-specific aspects from the case studies were investigated, also taking into consideration the perspective of local stakeholders. By integrating the results from each of the case studies we were able to develop a structured base of qualitative and quantitative data, and to analyse similarities and differences between cases, combinations and different categories of MU. This has provided a better understanding of the real needs for MU implementation in local contexts.

## Methods

In order to tackle these RIs, a methodology was defined which combined desk analysis with stakeholder engagement. This allowed for the exploitation of documented knowledge and data, as well as the collection of undocumented knowledge, and gathering of the most up-to-date stakeholder information. Due to the nature of the RIs considered, both qualitative (e.g. factors influencing MU development) and quantitative (estimation of MU Potential and MU Effect, as defined below) indicators were included in the analysis.

The following definitions were adopted:

**MU** = “In the realm of marine resource utilisation, multi-use should be understood as the joint use of resources in close geographic proximity" (i.e. in the same area). This can involve either a single user (one operator running two or more activities in the same location, e.g. an energy developer installing a offshore wind / wave hybrid platform) or multiple users (two or more distinct operators performing different activities in the same location, e.g. one offshore wind energy operator and one aquaculture operator). MU is an umbrella term that covers a multitude of use combinations in the marine realm and represents a radical change from the concept of exclusive resource rights to the inclusive sharing of resources by one or more users" [6].
**Resource** = a goods or service that represents a value to one or more users [6]. It refers to natural resources (e.g. fish stocks, ocean space, biodiversity rich ecosystems, etc.) as well as to human resources (staff), infrastructures (e.g. in the case of multi-use platforms), and money (e.g. costs for power, logistic, licensing procedures).
**Drivers** = factors promoting or strengthening the development of MU.
**Added values** = positive impacts of establishing or strengthening MU.
**Barriers** = factors hindering the development of MU.
**Impacts** = negative impacts of establishing or strengthening MU.
**DABI** = evaluation system considering Drivers, Added values, Barriers, Impacts.
**Potential MU** = no real (actual of past) examples of the combinations are found in the study area but opportunities for its development are identified (including planning and design phase).
**Implemented MU** = real example of the combination are available for the study area, even in pilot scale, or developed within a project.
**Hard use (H)** = a use of the sea requiring long-term installation of major infrastructures (e.g. platforms for offshore wind energy production or oil and gas extraction) [2].
**Soft use (S)** = a mobile and fleeting use, often requiring less investment and not demanding the realization of large infrastructures (e.g. recreation and tourism) [2].
The same use can be classified as hard or soft depending on the local context. An example of this is aquaculture: it can be implemented as a hard, industrial-scale, use (e.g. in the North Sea) or as a soft, traditional, small-scale use (e.g. in the Mediterranean).
**Nearshore (N)** = approximately <12nm.
**Offshore (O)** = approximately >12 nm.
**Traditional use (T)** = maritime sector / use of the sea traditionally established in the area, sometimes also linked to local jobs and traditions.
**New use (N)** = new maritime sector / use of the sea recently established or in process to be established in the area.
**Growing sector** = a maritime sector / use of the sea which is presently growing in terms of economic development, dimension.
**Static sector** = maritime sector / use of the sea which is presently static or declining in terms of economic development, dimension.
**Abiotic** = a maritime sector / use of the sea that is based on the exploitation of abiotic resource(s).
**Biotic** = a maritime sector / use of the sea that is based on the exploitation of biotic resources(s).

### Case study selection

**Fig 1:**
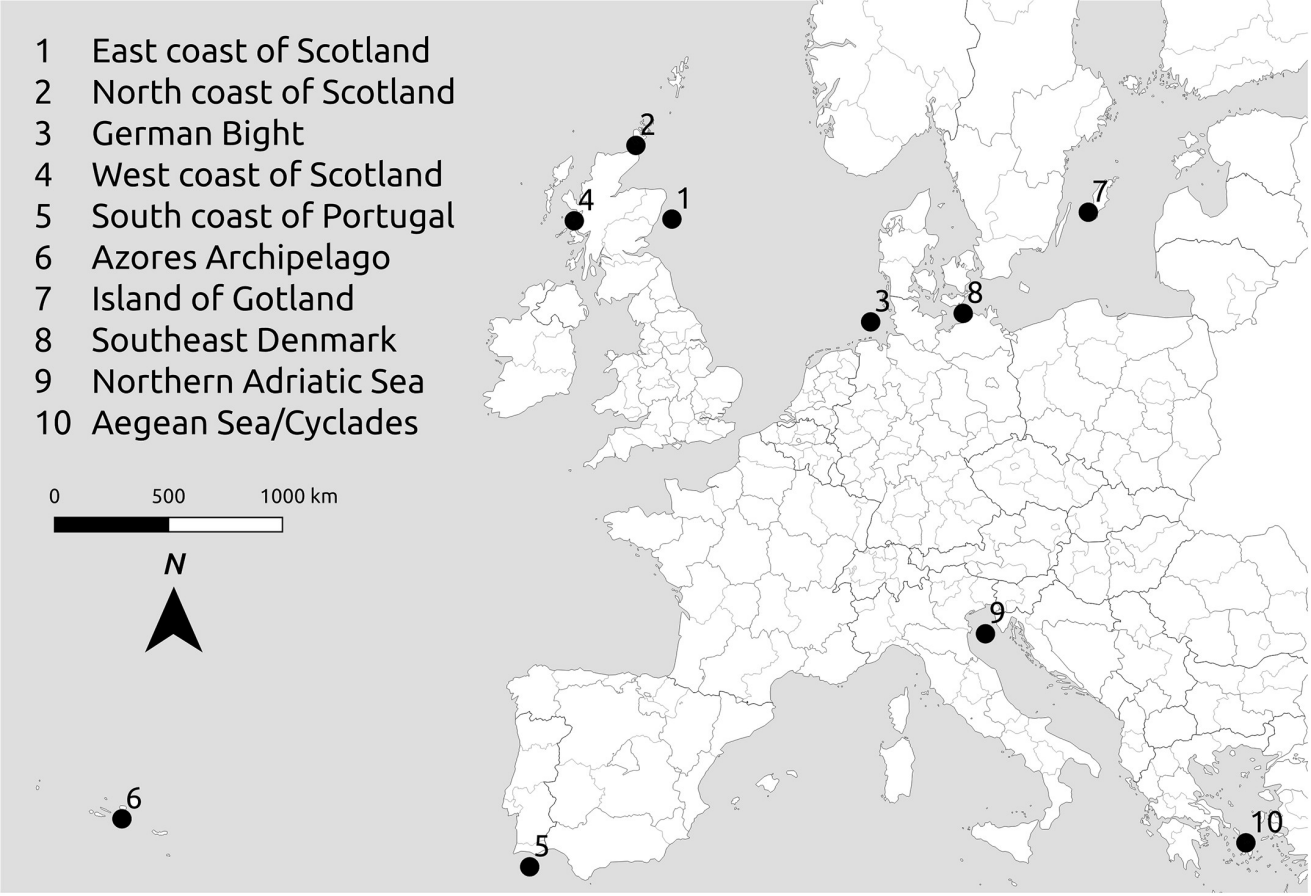
Geographical location of the 10 case studies (background map data from OpenStreetMap contributors). Case study 1 [27]; Case study 2 [28], [29]; Case study 3 [30]; Case study 4 [31]; Case study 5 [32]; Case study 6 [33]; Case study 7 [34]; Case study 8 [35]; Case study 9 [36]; Case study 10 [37].

In order to assess the most promising MU combinations for different specific contexts around Europe, to collect information on the various factors influencing MU, and to make recommendations for facilitating its development, ten case studies were selected (Fig 1).

For the selection of the case studies, the following criteria were considered:

i) Geographical representativeness of EU seas (Eastern Atlantic, North Sea, Baltic Sea, Mediterranean Sea).
ii) Offshore and nearshore activities representativeness.
iii) Coverage of different sectors of the blue economy.
iv) Consideration of both hard and soft uses.
v) Representativeness of different levels of implementation of the MSP process (from initial to fully mature).
vi) Representativeness of both implemented and potential cases of MU.

In relation to those MUs which are potentially implementable, we only considered cases where the involved sectors are already present, or are at the very least planned to be (as opposed to any MUs that could theoretically be conceived).

We included in the selection MUs which had already been investigated, as well as innovative combinations of emerging and traditional maritime uses. Less visible MUs which play a significant role in coastal communities were also included. In total, a wide variety of environmental and socio-economic conditions, as well as policy, research and technology development contexts, were brought into the analysis, with the aim of describing an extensive pattern of MU implementation and opportunities across Europe.

### Multi-use analysis in the case studies

**Fig 2:**
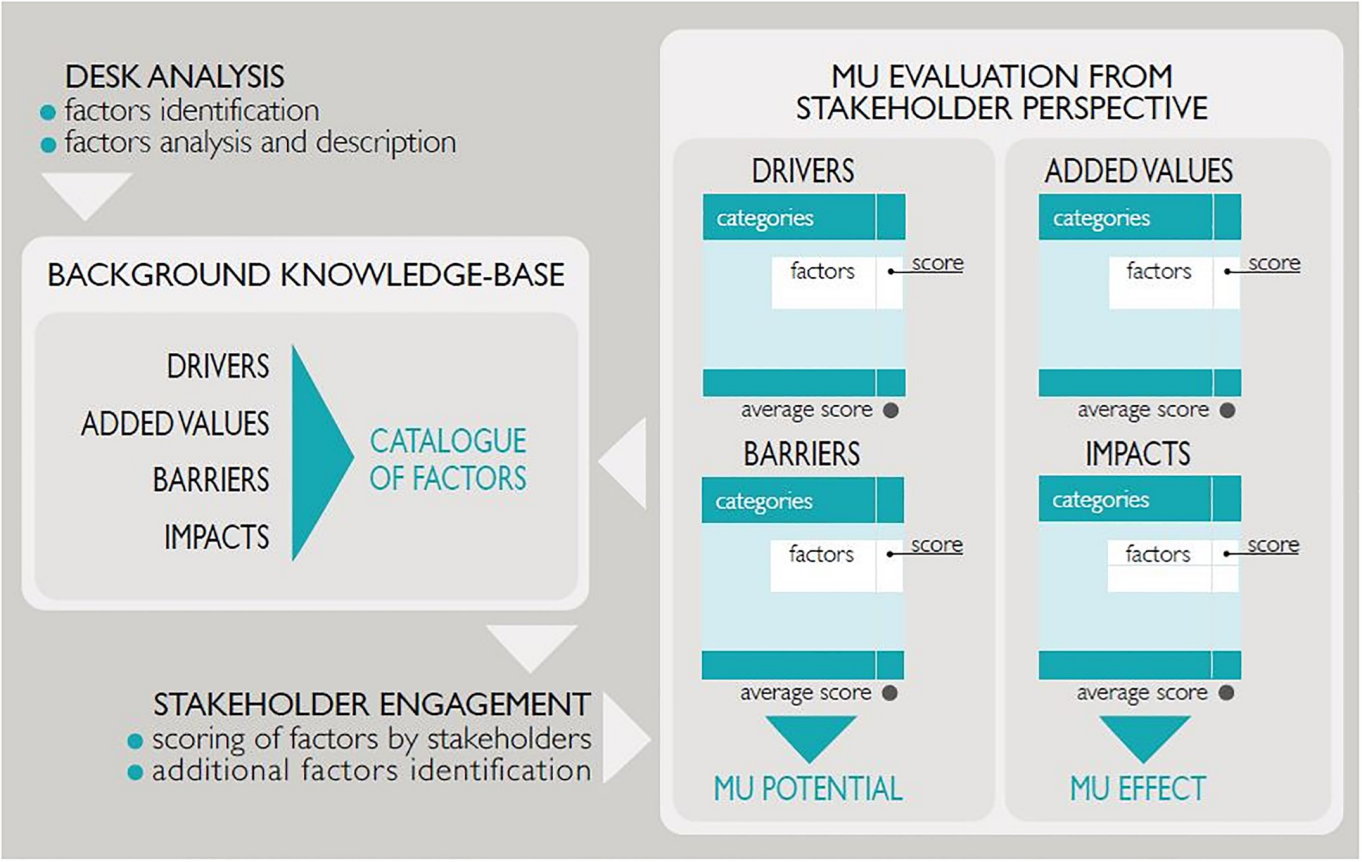
Conceptual scheme of the methodology used to analyse MU in the case studies.

The same approach and steps of analysis (Fig 2) were used in all case studies to ensure comparability. Desk analysis and stakeholder engagement were applied iteratively along the analysis process, with initial feedback from stakeholders resulting in additional desk research. Final feedback from stakeholders was collected at the end of the process. Stakeholders also provided reference to key documents to be considered in desk research.

#### Desk analysis

The aim of the desk analysis was to identify the following major aspects:

(i) characteristics of the study area (physical, environmental);
(ii) marine/maritime uses and activities (current status and trends focussing on those relevant for MU);
(iii) status of MU in the area, including background on existing and / or potential MU(s), information concerning legislation, institutional and administrative context at local level specific for MU or regarding the main economic sectors involved in MU, relevant national and / or local projects and experiences;
(iv) locations where MU is implemented, or planned, or where is the highest potential for certain MU combination(s), and;
(v) information Drivers, Barriers, Added values and Impacts (DABI) for each MU combination (identification of relevant factors).

For each DABI set, factors were further grouped into several categories: policy & legal, administrative, technical, other uses-related (e.g. drivers for MU linked to the need to reach/maintain good environmental quality), resources-related (e.g. drivers for MU linked to the presence of strategic infrastructures, like ports), risk & safety (e.g. barriers to MU linked to the need to satisfy safety criteria), economic, environmental and, social.

Data sources included: scientific literature (searched via on-line engines e.g. Google Scholar); policies, strategies, laws and regulations at national and sub-national levels; plans, environmental impact assessments (EIAs) and feasibility studies of in-progress or realized projects (primarily available at databases from local institutions and agencies), and national and international projects relevant for each case-study area. Information was also gathered from websites and documents available from economic operators, local agencies and other local stakeholders (e.g. FLAGS) in each case-study area.

For determining the cases of existing or potential MU developments, data sources were assessed and analysed to acquire information on the following elements:

i) type and aim of MU initiative,
ii) involved use(r)s (e.g. offshore wind, aquaculture),
iii) type of resources shared (e.g. biological, human, physical, geographical),
iv) order of development of MU (joint vs. staggered development),
v) geographic location,
vi) MU commencement (date),
vii) legal basis of MU (e.g. administrative obligation, private contract, research project),
viii) existence of funding to promote MU cooperation,
ix) key private/public actors for MU development,
x) level of maturity of MU (none, planned, design phase, full implementation, commercial use),
xi) Technology Readiness Levels (if applicable),
xii) advantages derived from MU (to singles uses, local communities, environment, etc.),
xiii) possibility of extension of the examined MU to include other uses.

Information was compiled in individual sheets, under respective categories in a tabular format, to enable acquisition of essential and consistent information across locations and projects, and show the comparison between MU and case-studies.

Results of desk analysis consist in: i) identification of the most promising MU combinations in each site, their current status, and perspectives for development (the most promising combinations included already implemented MUs and potentially implementable MUs); ii) compilation of catalogues of factors to analyse MU Potential (estimation of the degree of opportunity to develop or strengthen the MU) and MU Effect (balance of pros and cons of developing the MU) of each combination of uses; iii) compilation of a preliminary list of actions needed to support MU development.

#### Stakeholder engagement

The results of desk analysis were validated by stakeholder engagement. Engagement with stakeholders was undertaken in three steps:

i) to validate the identification of the most promising MU combinations;
ii) to score the DABI catalogues by the same or (partially) different group of stakeholders; and
iii) to identify actions to promote MU.

In some cases these three steps occurred in the same occasion (e.g. during a one single interview), in others over two different events (e.g. (i) during interviews and then (ii) and (iii) during a workshop).

Under step (i) the MU combinations preliminarily identified were discussed with the stakeholders and were then selected, revised or confirmed through their expert opinion. In some cases stakeholders suggested other MU combinations, not previously identified via the desk analysis.

Under step (ii) the preliminary DABI catalogues were evaluated and scored by stakeholders. Experts and stakeholders were also asked to identify additional factors, according to their knowledge/experience, which were included in the final DABI catalogue.

Finally, under step (iii) a preliminary list of actions to promote MU development was discussed with stakeholders and their input was used to update the list.

A scoring system was defined for DABI factors and quantitative definitions of MU Potential and MU Effect were introduced (Tables 1 and 2).

**Table 1:** Scoring system for drivers and barriers and definition of MU Potential.

**Steps to undertake in order to evaluate MU Potential**: - scoring of drivers by each stakeholder - calculation of the average of Drivers scores - scoring of Barriers by each stakeholder - calculation of the average of Barriers scores - MU Potential estimation.	
**Scoring of Drivers**: a positive sign is attributed to factors supporting MU according to the following scale: - high priority: score = +3 - medium priority: score = +2 - low priority: score = +1 - not relevant^1^: score = 0 - absent ^2^: score = 0 - I do not know ^3^: no score is given	**Scoring of Barriers**: a negative sign is attributed to factors hindering MU development according to the following scale: - high obstacle: score = -3 - medium obstacle: score = -2 - low obstacle: score = -1 - not relevant^1^: score = 0 - absent^2^: score = 0 - I do not know^3^: no score is given
**MU Potential** is evaluated by averaging the average of Drivers scores and the average of Barriers scores. MU Potential can assume values in the interval [-1.5, 1.5] ^4^, where -1.5 reflects an absolute negative MU Potential and 1.5 an absolute positive MU Potential. A value of 0 for MU Potential can occur when there is a balance between factors promoting MU development and factors hindering it. The development/strengthening of MU will therefore depend upon which of them will prevail. The knowledge of positive and negative factors is useful to address actions aimed at facilitating MU development.	

^1^ The factor is present, but it has no influence on MU Potential or MU Effect.

^2^ The factor is not present.

^3^ There is no knowledge about the factor

^4^ The negative extreme -1.5 is calculated by applying a score of -3 to all Barriers (B) and a score of 0 to all Drivers (D), calculating their averages, respectively (average of B = -3; average of D = 0), and finally calculating the average of these averages which is -1.5. The reverse process is applied for the positive extreme +1.5, where all Drivers were scored 3 and all Barriers were scored 0, and the average of the sum of their averages is +1.5 [38].

**Table 2:** Scoring system for added values and impacts and definition of MU Effect.

**Steps to undertake in order to evaluate MU Effect**: - scoring of Added values by each stakeholder - calculation of average of Added values scores - scoring of Impacts by each stakeholder - calculation of average of Impacts scores MU Effect estimation.	
**Scoring of Added Values**: a positive sign is attributed to factors representing benefits of developing or reinforcing MU and the following scoring scale is applied: - high added value: score = +3 - medium added value: score = +2 - low added value: score = +1 - not relevant^1^: score = 0 - absent^2^: score = 0 - I do not know^3^: no score is given	**Scoring of Impacts**: a negative sign is attributed to factors representing negative effects of developing or expanding MU and the following scoring scale is applied: - high impact: score = -3 - medium impact: score = -2 - low impact: score = -1 - not relevant^1^: score = 0 - absent^2^: score = 0 - I do not know^3^: no score is given
**MU Effect** is evaluated by averaging the average Added values score and the average Impacts score. MU Effect can assume values in the interval [-1.5, 1.5]^4^, where -1.5 reflects an absolute negative MU Effect and 1.5 an absolute positive MU Effect. The case of MU Effect = 0 can occur where there is a balance between pros and cons of MU development. The knowledge of positive and negative factors is very useful to address actions aimed at maximising the added value of MU.	

^1^ The factor is present, but it has no influence on MU potential or MU Effect.

^2^ The factor is not present.

^3^ There is no knowledge about the factor

^4^ The negative extreme -1.5 is calculated by applying a score of -3 to all impacts (I) and a score of 0 to all added values (A), calculating their averages, respectively (average of I = -3; average of A = 0), and finally calculating the average of these averages which is -1.5. The reverse process is applied for the positive extreme +1.5, where all added values scored 3 and all impacts scored 0, and the average of the sum of their averages is +1.5 [38].

Stakeholder engagement was performed for individual case studies, whereby local (municipality level) and regional (sub-national level) stakeholders and experts were selected according to their competences and expertise in each of the sectors involved in a potential MU.

A total of 125 stakeholders were engaged over the 10 case studies. Categories and numbers of stakeholder engaged are indicated in Table 3. The stakeholder engagement approach was decided on a case-by-case basis: all case studies used interviews and two of them (case study 4—Sweden and case study 9—Italy) also organised a workshop/focus group.

**Table 3:** Stakeholders engaged across cases. Stakeholders had the option to comment on more than one MU combination, therefore: total stakeholders = number of persons engaged; total contributions = number of inputs to DABI catalogues (factors identification and scoring).

Case study	Policy makers & regulators	Commercial sectors	Public-private partner-ships	Developers & consultants	Academics & experts	Civil society	Total stakeholders	Total contributions to DABI catalogues
1	-	6	-	-	3	-	9	9
2	5	2	6	5	4	-	22	36
3	1	1	-	1	1	-	4	8
4	5	3	-	2	-	1	11	15
5	3	4	-	-	1	1	9	11
6	5	3	-	-	1	3	12	16
7	2	2	-	2	3	2	11	14
8	3	1	-	5	1	1	11	17
9	5	8	2	1	6	2	24	29
10	5	1	-	1	1	4	12	19

Participating stakeholders had the option to comment on more than one MU combination and hence identify and score DABI factors for more than one MU, resulting in a total of 171 contributions to DABI catalogues.

All participants in the study signed the consent form prepared under the Horizon 2020 (H2020) Multi-use in European Seas (MUSES) project this study was part of. This consent form was prepared and developed under the Ethical Requirements held in the Grant Agreement (Work Package 6 of the project) to ensure compliance with EU H2020 funded projects. The stakeholder consent form and the Informed Consents Procedures Manual (also developed under Work Package 6) that partners have followed during the course of the study were approved by The Innovation and Networks Executive Agency (INEA), the funder for MUSES.

### Data elaboration

An integrated DABI catalogue was built based on the results from the 10 case studies and including all factors identified (about 1000 rows), the scores attributed by stakeholders and several descriptive attributes. Statistics and graphics presented in this publication were computed using Python code and organised in a Jupyter notebook, fully available in [39].

In order to cluster MU combinations into groups, to be analysed and compared through further analysis, several general characteristics of MU combinations were defined, as described in Table 4.

**Table 4:** Characteristics used to describe the MU combinations (for words in bold see the definitions given in the methods).

Characteristic	Definition
Driving sector	It is the strongest maritime sector/use of the sea included in the MU combination, from the economic point of view and/or in terms of dimension (e.g. number of activities, number of employees, etc., in the specific case study).
Implementation	Two alternative options are used to classify the combination, according to its implementation level: **Potential** and **Implemented**.
Type	It relates to the infrastructure type necessary for the uses of the sea involved in the combination. **Hard uses (H)** and **Soft uses (S)** are considered. Three alternative options are used to classify the combination, according to its type: Hard&Hard, Hard&Soft, Soft&Soft.
Location	Two alternative options are used to classify the combination, with reference to the sea area for actual or potential location of MU: **Nearshore** and **Offshore**.
Innovation	**Traditional uses** and **New uses** are considered. Three alternative options are used to classify the combination, according to its level of innovation: Traditional&Traditional, Traditional&New, New&New.
Trends	**Growing** and **Static** uses are considered. Three alternative options are used to classify the combination, according to the trends of the involved sectors: Growing&Growing, Growing&Static, Static&Static.
Resources	Uses engaged in the combination can exploit natural **Abiotic** or **Biotic** resources. Tree alternative options are used to classify the combination, according to the nature of the resources exploited: Abiotic&Abiotic, Abiotic&Biotic, Biotic&Biotic.

A coefficient of variation (CV) was associated to MU Potential and MU Effec in order to take into consideration variability of the given scores. CV is calculated as the ratio of the standard deviation σ to the mean μ. For example, in the scenario case of the CV related to MU Potential (P), calculated using Drivers (D) and Barriers (B) scores, the following formula applies:

$${CV}_{P}=\left(\frac{\sigma_{D}}{\mu_{D}}-\frac{\sigma_{B}}{\mu_{B}}\right)/2$$

Information gathered during the desk analysis and stakeholder engagement process was compiled and analysed according to the RIs:

**For RI-1**: the most promising MU combinations for each case study were identified (by desk analysis and stakeholder opinion) and described according to the characteristics defined in Table 4. With this analysis we can provide a picture of: what MU consists, or could consist, of in different European locations; where MU is, or could be, developed (inshore or offshore).
**For RI-2 and RI-3**: MU Potential, MU Effect and DABI results were analysed by (i) combinations and, (ii) by cases. Here we highlight the combinations scored by stakeholders as the most promising (easy to implement or to strengthen) and those scored as the most beneficial (considering a set of economic, societal and environmental factors). We also provide insights on the categories of factors determining these results, presenting a examples of specific DABI factors from the different cases.
**For RI-3**: MU Potential and MU Effect were analysed by grouping combinations according to some of general characteristic of Table 4, namely Location, Type and Driving sector. With this, we provide the basis for some more general considerations about MU development by identifying some characteristics of MU that are considered more promising and more beneficial.
**For RI-4**: actions recommended by stakeholders to facilitate and promote MU implementation and strengthening were organised in categories, according to the type of action suggested (results presented in 3.5). With this analysis, we indicate some actions that could be considered at local, national and EU level to confidently promote MU development.

## Results

### Most promising MU combination in specific European location

16 most promising MU combinations were identified over the 10 cases studies (Table 5).

**Table 5:** MU combinations identified as most promising in the case studies and their major characteristics.

	Case Study No.	MU Combination	Driving Sector	Implementation	Type	Location	Innovation	Trends	Resources	#DR	#BA	#AV	#IM	No. DABI Factors (Total)
1	1	WI & F	WI	Potential	Hard&Soft	Offshore	Traditional&New	Growing&Static	Abiotic&Biotic	19	17	10	19	65
2	2	TI & E	TI	Potential	Hard&Soft	Nearshore	Traditional&New	Growing&Static	Abiotic&Biotic	9	11	6	17	43
3	2	TI & M	TI	Potential	Hard&Hard	Nearshore	Traditional&New	Growing&Growing	Abiotic&Biotic	8	1	10	0	19
4	3	WI & F	WI	Potential	Hard&Soft	Offshore	Traditional&New	Growing&Static	Abiotic&Biotic	4	4	3	1	12
5	3	WI & A	A	Potential	Hard&Hard	Offshore	New&New	Growing&Growing	Abiotic&Biotic	3	7	3	1	14
6	4	WA & A	WA	Potential	Hard&Hard	Both	New&New	Growing&Growing	Abiotic&Biotic	33	35	23	9	100
7	4	S & R	S	Potential	Hard&Hard	Nearshore	Traditional&New	Growing&Static	Abiotic&Abiotic	24	36	10	6	76
8	5	T & A	T	Implemented	Soft&Soft	Nearshore	Traditional&Trad.	Growing&Growing	Biotic&Biotic	23	24	20	2	69
9	5	T & E	T	Implemented	Soft&Soft	Nearshore	Traditional&Trad	Growing&Static	Biotic&Biotic	12	9	12	4	37
10	5	T & F	T	Implemented	Soft&Soft	Nearshore	Traditional&Trad	Growing&Static	Biotic&Biotic	19	15	13	1	48
11	6	T & F	T	Implemented	Soft&Soft	Nearshore	Traditional&Trad	Growing&Static	Biotic&Biotic	25	17	20	1	36
12	6	T & H & E	T	Implemented	Soft&Soft	Nearshore	Traditional&Trad	Growing&Growing	Abiotic&Abiotic	21	7	13	5	46
13	6	T & E	T	Implemented	Soft&Soft	Both	Traditional&Trad	Growing&Growing	Biotic&Biotic	11	4	6	4	27
14	7	WI & A	WI	Potential	Hard&Soft	Offshore	New&New	Growing&Static	Abiotic&Biotic	8	10	6	5	29
15	7	WI & T	WI	Implemented	Hard&Soft	Offshore	Traditional&New	Growing&Static	Abiotic&Abiotic	8	8	10	5	31
16	8	WI & A	WI	Potential	Hard&Soft	Offshore	New&New	Growing&Growing	Abiotic&Biotic	11	20	11	15	57
17	8	WI & E & T	WI	Potential	Hard&Soft	Offshore	Traditional&Trad	Growing&Static	Abiotic&Biotic	17	24	15	13	69
18	9	T & F	T	Implemented	Soft&Soft	Nearshore	Traditional&Trad	Static&Static	Biotic&Biotic	10	12	10	4	36
19	9	T & A	T	Potential	Soft&Soft	Nearshore	Traditional&Trad	Static&Static	Biotic&Biotic	9	10	9	4	32
20	9	T & E	T	Potential	Soft&Soft	Nearshore	Traditional&Trad	Static&Static	Biotic&Biotic	9	13	9	4	35
21	9	T & H	T	Potential	Soft&Soft	Nearshore	Traditional&Trad	Static&Static	Abiotic&Abiotic	10	10	5	4	29
22	9	O&G & T & A	O&G dec.	Potential	Hard&Soft	Offshore	Traditional&New	Growing&Static	Abiotic&Biotic	10	22	42	7	51
23	9	O&G & R	O&G dec.	Potential	Hard&Hard	Offshore	Traditional&New	Growing&Static	Abiotic&Abiotic	7	24	15	8	54
24	10	R & D	R	Potential	Hard&Hard	Offshore	New&New	Growing&Growing	Abiotic&Abiotic	8	15	13	0	36
25	10	T & F	T	Implemented	Soft&Soft	Nearshore	Traditional&Trad	Growing&Static	Biotic&Biotic	8	7	12	0	27

Acronyms used in the table: WI = offshore wind energy production; TI = tidal energy production; E = environmental protection; M = environmental monitoring; F = fisheries; A = aquaculture; WA = wave energy production; S = shipping terminal; T = tourism; H = underwater cultural heritage; O&G = oil & gas decommissioning; R = marine renewable energy production (this term indicates the production of energy from renewable sources in general, with no reference to a specific energy source); D = desalination; DR = Drivers; BA = Barriers; AV = Added Values; IM = Impacts.

Combinations were classified according to the characteristics given in Table 4, with reference to each case study and the local context where MU was analysed. Other, less relevant combinations were identified in some of the case studies but were not considered for further analysis due to their comparative immaturity or lack of significant interest from stakeholders.

As 5 combinations (out of the 16 most promising ones) were identified in more than one case, Table 5 includes 25 rows, in order to illustrate the characteristics of all combinations in all cases. Brief description of the combinations is provided in Table 6.

**Table 6:** Brief description of the combinations identified as most promising.

Combination	Description
WI&F	MU combination between offshore wind energy production and commercial fisheries. Two case studies explored this combination, both located in the North Sea (1—East coast of Scotland, 3—German Bight). Wind farms with fixed foundations in combination with commercial fisheries (mobile and static gears) represent the main focus of case study 1, while results are directly transferable to emerging floating offshore wind and hybrid platform markets. Wind farms with fixed foundations are also considered in case study 3, where the offshore wind energy sector is reported as a relatively new sector that is poised to become one of the major sectors vying for space due to its exponential expansion in recent years. The two sectors compete for space since they both seek access to locations which share the same physical characteristics (examples of scallop dredging and langoustine trawling were explored in case 1).
TI&E	This combination was investigated in case study 2 (North coast of Scotland). Tidal current turbines and environmental protection areas can be co-located in order to maximize spatial efficiency, where significant adverse environmental impacts, and/or impacts on the local and regional economies, can be excluded, or where advantageous environmental, economic and social synergies can be shown. Environmental protection areas can include different regimes of protection encompassing Special protected areas (SPAs), Special Areas of Conservation (SACs), Marine Protected Areas (MPAs), Sites of Specific Scientific Interest (SSSIs), and locally designated sites.
WI&A	This MU envisages the combination between offshore wind energy production and different types of aquaculture (shellfish, finfish, seaweed). Three case studies addressed this combination: case 3 in the German Bight considered combination with aquaculture in general, case 7 looked at the Island of Gotland (Sweden) and the idea of using the existing piles of the wind park to attach longlines for mussel farms, and case 8 in Southern Denmark considered combination with mussels/seaweeds. According to the North Sea experience, offshore aquaculture installations within the priority area for offshore wind energy production might be implemented through: (i) the direct attachment of installations, such as cages or long-lines, to offshore wind turbine foundations, or through; (ii) the co-location of aquaculture installations within the security zone of the offshore wind energy production. The first option was assessed as being not possible for the wind farms currently in operation, or for those already licensed, because complex engineering adjustments are needed in the planning phase to accommodate an extra load within safety margins. Similarly, case study 8 implies co-location with existing wind farms (sharing space, equipment, services) rather than infrastructural integration.
TI&M	This combination was investigated in case study 2 (North coast of Scotland) and explores the potential for integrating various types of monitoring equipment such as passive acoustic, sonar, audio and visual on a MU platform, and co-locating such equipment on tidal current turbine structures.
S&R	This MU was investigated in case study 4 (West coast of Scotland). The MU involves the generation of green energy from marine renewable sources in general (offshore wind, wave and tide), its transmission to a port substation and the potential to use energy to cover the requirements of the port, in addition to other benefits (e.g. Green-House Gas (GHG) reductions and human health benefits). The potential to use the energy to power auxiliary engines of berthed vessels (shore-side electricity) was also investigated.
T&A	This combination was explored in two case studies, one located in the Southern coast of Portugal (case study 5) and the other located in the Northern Adriatic Sea (case study 9). In case study 5, aquaculture facilities are used as potential tourist attractions where recreational activities, including diving, are developed. Alternative or integrated ways to combine aquaculture and tourism have been identified for the case study 9 area: boarding of people on aquaculture vessels to visit sea farms and learn aquaculture techniques for educational and recreational purposes; sport fishing tourism (mainly angling) next to mussel aquaculture plants which commonly function as attractive marine areas for a number of fish species; diving/snorkelling tourism, which could be practiced next to aquaculture farms, where a rich fauna can be observed.
T&E	This combination was addressed by three case studies located in the Eastern Atlantic Basin (case 5, South coast of Portugal, and case 6, Azores archipelago) and in the Mediterranean (case 9, North Adriatic). It consists of the development of touristic activities (mainly diving) inside designated MPAs, managed with the goal to preserve natural resources. It is also seen as an opportunity to expand the protection of the marine environment, while at the same time developing socio-economic activities, with advantages for both sectors. The implementation of this MU would require the establishment of links between tour operators, touristic service providers, institutions and associations involved in the field of marine protection, highlighting potential mutual advantages. According to the results of case study 9, this MU could be promoted also through a connection with the related environmental/naturalistic touristic activities on land (e.g. land-based facilities dealing with protection and recovery of specific marine species).
T&F	This was identified in four case studies, two of them located in the Eastern Atlantic Sea basin (case 5, South coast of Portugal, and case 6, Azores archipelago) and two in the Mediterranean Sea basin (case 9, North Adriatic Sea, and case 10, Aegean Sea). In all of these, the combination is described as “pescatourism”, which can be generally defined as the boarding of people, which are not part of the crew, onto small scale fishing boats for recreational and cultural purposes. Professional small-scale fishers play a central role in promoting and educating tourists on the environmental, socio-cultural and economic values of coastal areas by showing them fishing techniques, as well as offering or cooking local food on board. Pescatourism must not be confused with “recreational fishing”, “angling” or “sport fishing” which do not involve fishery operators, know-how, or boats of professional fisheries.
T&H&E	This MU triplet was explored in case study 6, for the Azores Archipelago. It is characterizsed by tourist and recreational activities developed in UCH sites, where environmental measures are also established. According to this combination, UCH benefits from the conservation management measures of environmental protection areas, with tourism benefits from both sectors.
WI&T	This combination involves the possibility to develop touristic activities in or around offshore wind energy production areas. This combination, which was addressed by one case study located in the Baltic Sea (Island of Gotland, case study 7) considers various examples: creating artificial sites for seals, boat tours that include information on renewable energy systems, or even creating art installations on the monopiles, potentially in combination with light and/or water shows. Recreational fishing boat tours to the wind farm was also considered, although further research is needed to explore the possible negative effects of noise generated by offshore wind energy production to large fishes.
WI&E&T	This MU triplet was considered in case study 8 (Southern Denmark), where touristic activities in and around offshore wind energy production areas could include diving and environmental education initiatives. The establishment of artificial reefs within the wind park located in the case study area might recreate marine environments that would otherwise have been eliminated as a result of historical stone dredging, thus encouraging the establishment of various marine species, increasing biodiversity, which would support this new form of tourism.
T&H	This combination was identified as the most promising in case study 9, for the area of the Northern Adriatic Sea (Mediterranean Sea basin). It involves the touristic exploitation of UCH sites (wrecks), specifically through diving activities, with the aim of valorising and safeguarding the cultural heritage from the current risk of looting and damage. This combination was also considered, in addition with environmental protection, in the MU triplet described here above.
O-G&T&A	This combination was identified among one of the most promising for case study 9 (Mediterranean Sea). The case study specifically took into consideration the projected decommissioning of 21 platforms by 2021–2022 in the Adriatic Sea (8 in the case study area), and, by extension, the need to identify potential re-uses of the dismissed infrastructures. This combination refers to a decommissioned O&G platform re-used to support recreational activities (e.g. diving, recreational fishing, environmental education, marinas, gastronomic experience) and functioning as structural and/or logistical support for aquaculture installations.
O-G&R	This combination was identified in case study 9 (see previous point). In this case, decommissioned platforms can be used for supporting renewable energy devices for wave, wind energy, and solar energy generation.
R&D	This combination was identified in case study 10, in the Mediterranean Sea basin (Aegean Sea—Greece). The main focus of this case study was to examine the possibility of installing offshore marine renewable energy and desalination platforms (i.e. energy production and desalinated water production), considering that the island of Mykonos has increased energy needs, as well as high quality freshwater demands during the high tourist season. The island has unique sustainable resources (wind, solar, wave) that could supply renewable energy systems.

Amongst the 16 different MUs identified, four are currently implemented and 12 have potential to be developed in the future within the specific sites. Most MUs involve two sectors (MU pairs), while the remaining three combinations envisage synergies amongst three different sectors in the same marine space (MU triplets). Seven combinations are to be located offshore, seven nearshore, and two in both contexts. A high degree of heterogeneity and local specificity in terms of combinations is shown across the case studies, with 11 combinations identified in only one case study area and five combinations identified as the most promising for more than one case study. A total of 13 maritime sectors were considered in the MU combinations, with tourism, aquaculture and offshore wind energy showing a higher propensity towards accommodating MU, being identified in a larger number of combinations. Tourism, aquaculture and fisheries, followed by environmental protection and offshore wind energy, were the sectors considered by a greater number of case studies. Renewable energy production at sea—either addressing a specific source (wind, wave, or tidal) or interpreted collectively—is considered in 10 of the 16 combinations explored.

A total of 25 DABI catalogues were compiled across the 10 case studies, with 23 being scored. Only the two catalogues for the combinations with O&G decommissioning in case study 9 were not scored. For this reason the O&G decommissioning combinations do not appear in the following figures. Each catalogue consists of four tables, one for each DABI group. The overall DABI catalogue for the complete 16 MU combinations is freely available in [40].

### MU analyzed across combinations (MU Potential, MU Effect and DABI)

#### MU Potential and Effect

**Fig 3:**
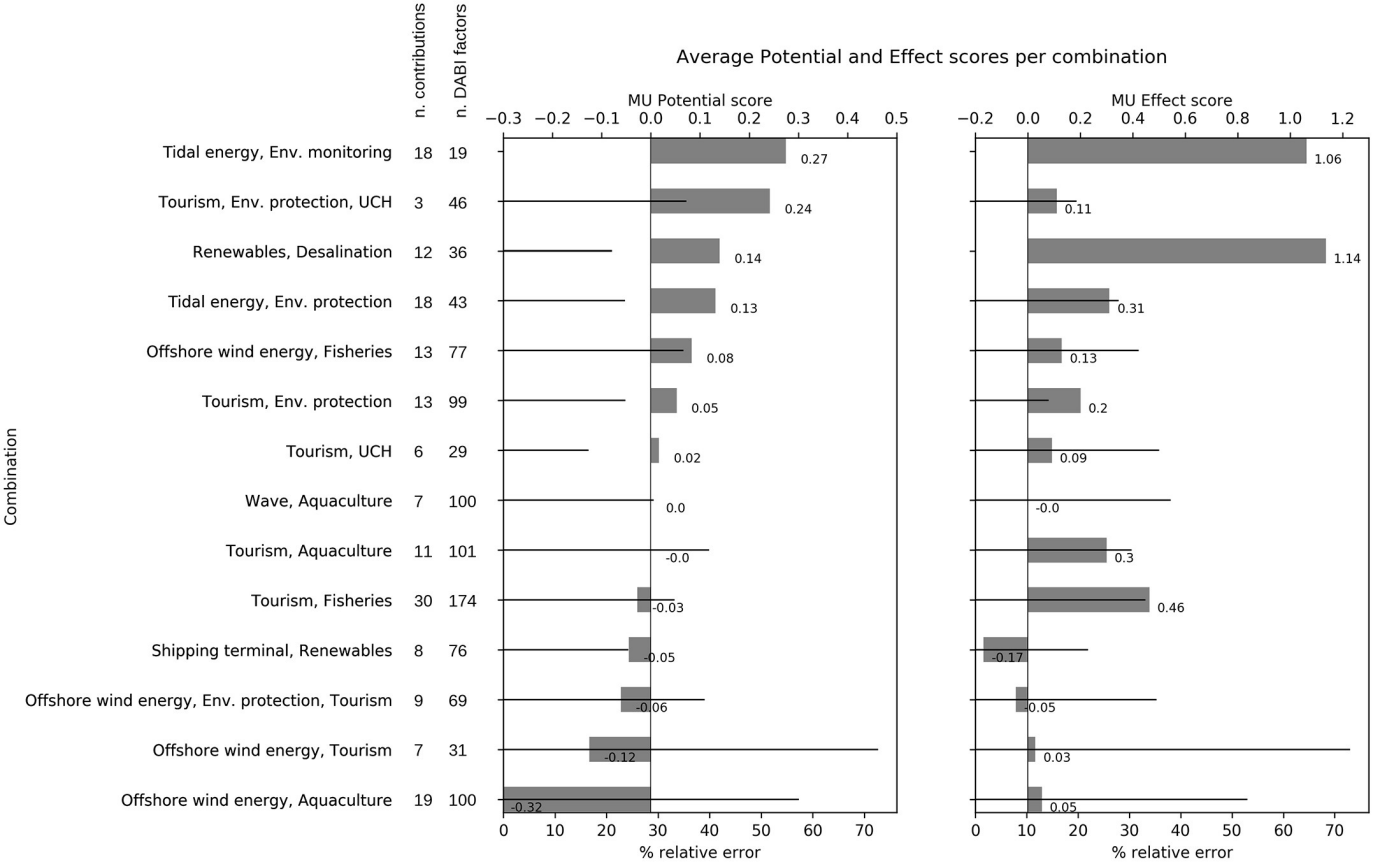
Average MU Potential and MU Effect for combinations and total number of DABI factors identified by stakeholders.

When comparing MU Potential for the various combinations (Fig 3—left), we note that approximately half of the combinations are associated with a positive value for MU Potential and the other half negative.

The combinations with the highest MU Potential are TI&M (0.27), T&E&H (0.24) and R&D (0.14). However, there was a tendency for MU potential values to score very close to 0, ranging between -0.3 and +0.3. Such results are determined by a balance between the average score of Drivers and Barriers provided by stakeholders’ opinions. For all combinations, these two elements are scored highly (average score of Drivers ranges between: 0.4 and 2.5; average score of Barriers ranges between: -0.7 and -2.5). The generally high average Driver scores suggest that interviewed stakeholders perceive there to be opportunities for MU development (i.e. MU implementation or strengthening/widening). Moreover, removing or decreasing the main Barriers can lead to the creation of conditions favourable for the development of MU. The values of the CV show a variability for the average of MU Potential around or below 30% for most combinations. Instead, MU Potential for WI&T and WI&A is associated with high values of CV, derived from very heterogeneous scores assigned by stakeholders, affecting the significance of average MU Potential estimation.

MU Effect reveals more homogeneity and was generally estimated as positive by stakeholders (Fig 3—right), with only two combinations (WI&E&T, S&R) having negative impacts prevailing over Added Values. MU Effect ranges from -0.2 to 1.1, assuming higher values than those associated to MU Potential. The combinations showing the highest MU Effect are R&D (1.14) and TI&M (1.06), also scoring amongst the highest for MU Potential. In fact, all combinations with positive MU Potential show also positive MU Effect, demonstrating coherence in the method and response by stakeholders. The average score of Added Values and Impacts was in a similar range (0.4–2 and -0.34–-2.6 respectively) but with the prevalence of Added Values.

For MU Effect, the values of CV show a higher variability, with values around or below 40% for most of combinations. Similarly for MU Potential, MU Effect for WI&T and WI&A is associated with high values of CV, significantly affecting the estimation of average MU Effect.

Despite the balance between Drivers and Barriers identified in all combinations, there seems to be a common agreement between stakeholders to consider MU as beneficial, if or when it is implemented, with Added Values prevailing over Impacts.

#### DABI analysis

**Fig 4:**
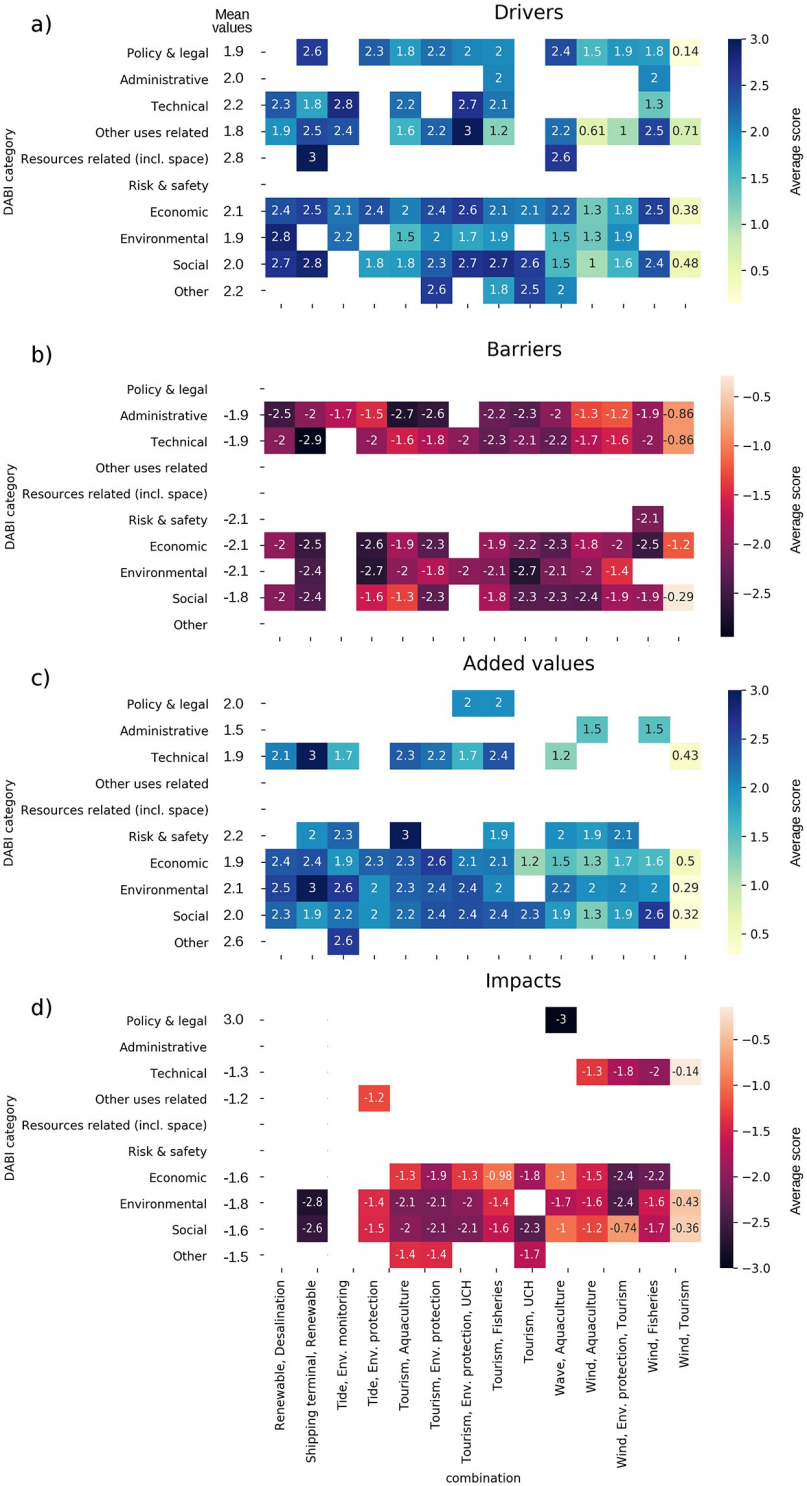
Average scores according to stakeholders for different categories of (a) Drivers, (b) Barriers, (c) Added Values, and (d) Impacts across all combinations.

Results of the scored DABI catalogues are represented in Fig 4, depicting the average Driver, Barrier, Added Value and Impact scores per combination.

Different categories of **Barriers** are scored similarly across MU combinations (Fig 4(b)). For example barriers average score was-1.9 for Technical barriers, -2.1 for Economic barriers, -1.9 for Administrative barriers, and -1.9 for Social barriers. Administrative barriers are particularly relevant for R&D and for the combinations with tourism (concessions of permits, licence limit and complex bureaucratic procedures). For example, in case studies 5 (Portugal/Algarve) and 9 (Italy), the lack of a common regulation at national levels for aquaculture-related tourism activities, restrictive rules concerning the number of people hosted aboard aquaculture vessels, and/or hygiene and security constraints, were highlighted as relevant barriers for T&A.

Barriers related to social acceptance are relevant for some of the combinations involving aquaculture (WI&A, WA&A). Important factors identified under this were: limitations to cooperation and dialogue among sectors; scarce public awareness of positive implications of MU, and; limited knowledge of cumulative environmental impacts of the combination. This is also true for T&E and T&H. In these cases, lack of social acceptance is related to resistance from underwater cultural heritage authorities and environmental non-governmental organizations (NGOs) due to fear of looting/damage of artefacts and damage of natural ecosystems.

Generally, the combination most limited by barriers is S&R (average score = -2.54), followed by T&H (-2.35) and WA&A (-2.2), while the less impacted are W&T (-0.68), followed by WI&E&T (-1.70) and TI&M (-1.73).

According to stakeholders, almost all combinations are associated to Economic, Environmental and Social **Added Values** with a comparable average score of 2.0–2.1 (Fig 4–4c). Technical Added Values are particularly relevant for S&R (e.g. in case study 4—West Scotland, MU can provide availability of proof of concept upon which more effective and cheaper global solutions can be based); benefits concerning risk management and safety are envisaged from the combination of T&A (e.g. in case study 5—Portugal/Algarve, MU can provide sharing of responsibilities and safety-related costs); Environmental Added Values are foreseen for S&R (e.g. in case study 4, MU can provide a reduction in GHG emissions where shipping terminals tend to be emission hot spots), R&D (e. g. in case study 10,—Greece MU can provide a low-carbon footprint of energy/water supply), TI&E (e.g. in case study 2—North Scotland, in addition to reduction of GHG emissions, turbine supports may create artificial reefs and the overall areas will likely act as no-fishing zones).

Numerous Added Values are expected from the implementation of the T&F combinations: Economic Added Values, since pescatourism can produce an additional income for fishers due to the development of new market opportunities and sector diversification, as well as benefits for the local economy, through an expected increase of commercialisation of local fish products; Social Added Values are expected for this combination since case studies recognized a general professional growth of operators involved in pescatourism, with the improvement of personal skills and management capacity. Educative benefits for tourists and civil society are also expected, with an increased awareness about issues related to the marine environment and fisheries. In case studies 5, 6, 9 and 10, Environmental Added Values include the possible contribution of pescatourism to the sustainable management of fisheries and the relief from coastal tourism pressures.

From a general perspective, the combinations that seem to have more benefits (higher Added value) to provide are T&E (average score = 2.42), T&A (2.31), S&R (2.30), followed by R&D (2,27), T&F (2.19) and T&H (2.1).

### MU analyzed across case studies (MU Potential, MU Effect and DABI)

#### MU Potential and Effect

**Fig 5:**
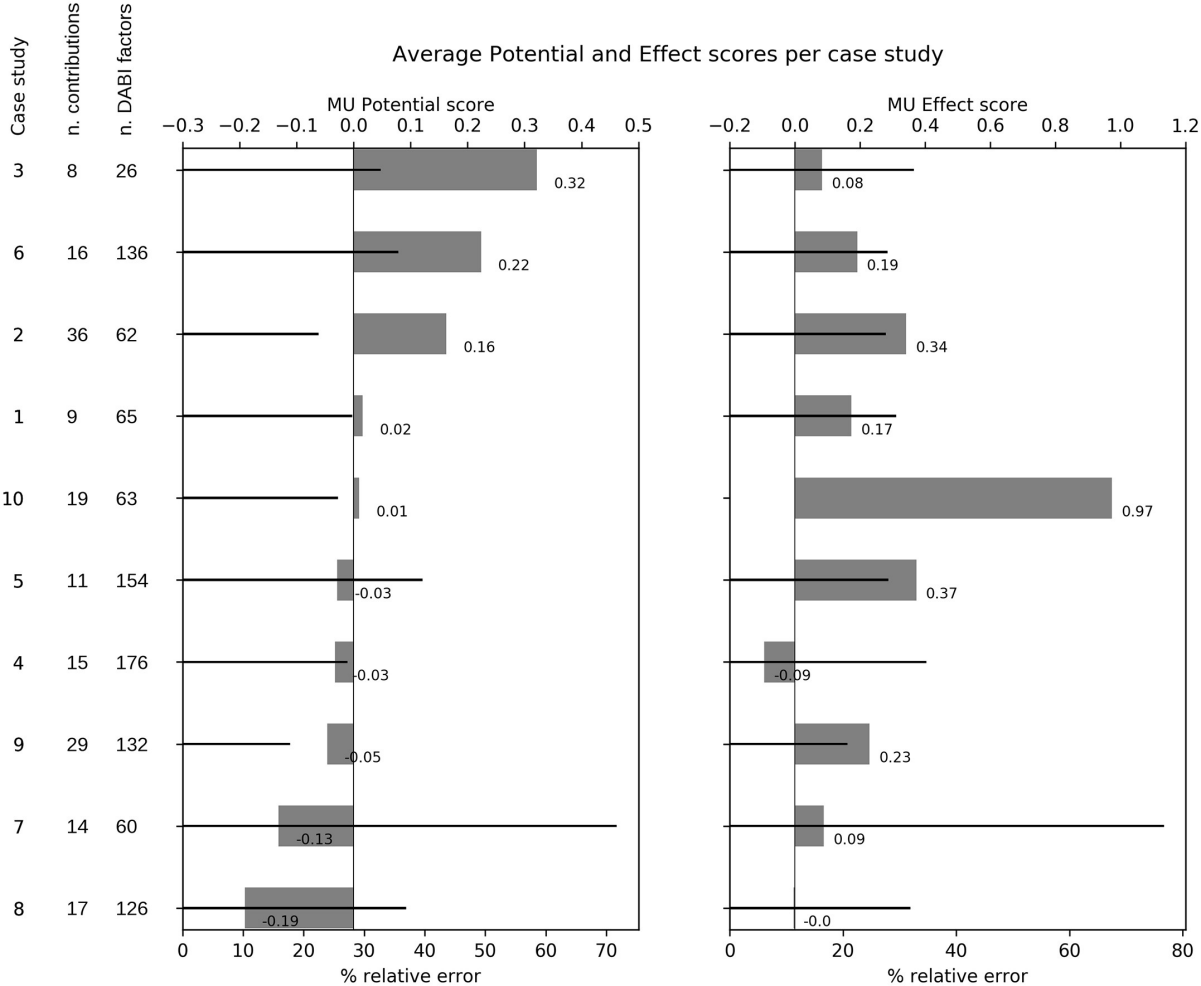
Average MU Potential and MU Effect provided by stakeholder perception in the 10 case studies (results from all combinations considered in each case are averaged together), and total number of DABI factors identified by stakeholders.

MU Potential shows positive values in half of the case studies and negative values in the other half, with all results close to 0 (Fig 5).

The 3 MUs with the highest potential are found in case study 3, considering the potential development of MU between WI&F and WI&A in the German EEZ; in case study 6, considering the potential development of tourism driven MUs (T&E, T&F, T&H&E) in the Azores; and in case study 2, considering the potential development of tidal energy driven combinations (TI&E, TI&M) in North Scotland. The CV associated to MU Potential in these three cases ranges between 25–35%, which demonstrates general agreement in the scores expressed by stakeholders. The cases with the lowest MU Potential are case study 8 (considering the potential development of MU in Denmark between WI&A and WI&A&T); case study 7 (considering WI&A and WI&T in Sweden); and case study 9 (in Italy, considering four combinations with tourism: T&F, T&A, T&E, T&H). Case study 7 shows a very high CV (70%), indicating a high degree of disagreement in the scores attributed by different stakeholders.

All cases with a positive MU Potential show a positive MU Effect as well. Some of the cases indicating negative MU Potential values are associated to positive values for MU Effect (cases 5, 7, 9), demonstrating stakeholder’s positive expectations for MU development. Eight cases in total show a positive MU Effect and the other two show results very close to 0. The highest estimated MU Effect is associated to case 10 (Greece) where two different MUs were considered: (R&D and T&F).

#### DABI analysis

**Fig 6:**
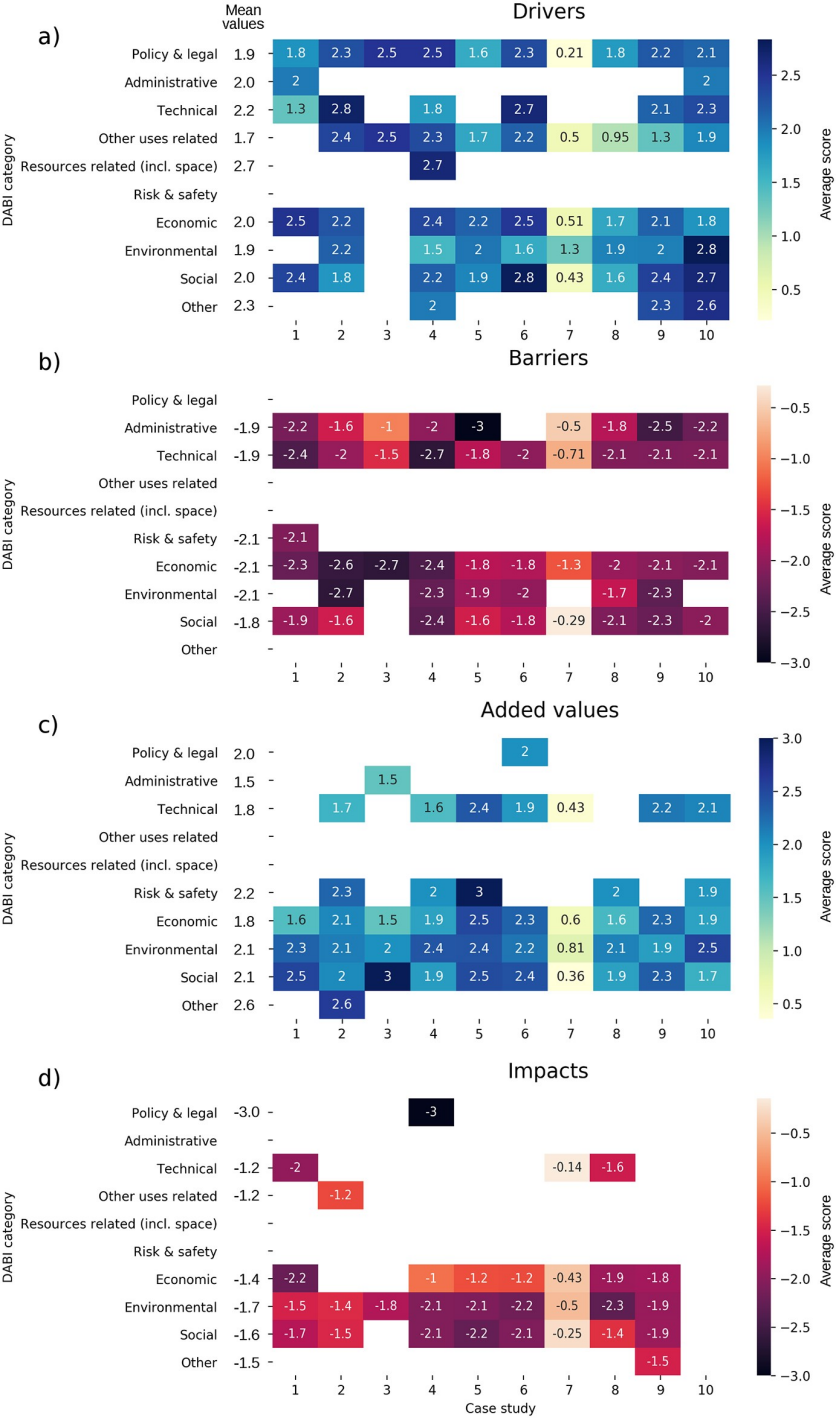
Average scores according to stakeholders for different categories of (a) Drivers, (b) Barriers, (c) Added Values, and (d) Impacts across cases.

Results of the scored DABI catalogues are represented in Fig 6.

For **Barriers** (Fig 6b), the average scores of different categories are very similar or equal: Economic, Risk & Safety and Environmental average scores are all equal to -2.1. Despite this, the distribution across the case studies highlights some interesting attributes. Differences in the importance of Administrative barriers can be highlighted; these are very relevant for case studies 5 (3.0), 9 (-2.5), 1 (-2.2) and 10 (-2.2). For example, in case 5 (Portugal/Algarve) the complexity of the licensing procedure (including the need for holding a second licence to practice MU) was highlighted for T&A and T&F. A lack of communication/coordination among the authorities dealing with UCH and tourism was also reported for case 9 (Italy). Issues related with the consultation process between the two sectors (timing, frequency, lack of support, governance structure, representation and, power imbalances) were mentioned for WI&F in case 1 (East Scotland). In case study 10 (Greece), a factor possibly blocking the operational stage of R&D was a need for consensus for multiple administrative and private interests. These examples demonstrated the role of the local context in determining conditions more or less favourable to MU.

Technical barriers show heterogeneity of scoring across cases in relation to the specific MU combinations, with the highest values related to case study 4 in the West of Scotland (e.g. difficulties in offshore wind energy transmission and storage in ports; a lack of available infrastructure for Shore Side Electricity (SSE) in ports; and that it is impractical to convert some types of vessels like tankers and cargo to new powering systems), and case study 1 in the East coast of Scotland (e.g. incompatibility of offshore wind energy components with fishing operations and vice versa; and fishing vessels and gears not compatible with altered sea conditions due to presence of offshore wind farms).

Conversely, Economic and Social barriers show a more homogeneous distribution across the cases.

Considering **Added Values** (Fig 6c), economic benefits are expected particularly in case studies 5, 6, and 9, deriving from the implementation of tourism-driven combinations (e.g. increase of revenues at local levels, cost reduction, diversification of tourism sector). Environmental Added Values from MU implementation are expected across all cases (e.g. WI&F in case 1—East Scotland where wind farms provide nurseries and sheltered areas contributing to strategic fisheries management as marine protected areas; WI&A in case 7—Sweden where mussel farms or seaweed cultivation can increase nutrient uptake and reduce eutrophication impacts; T&H in case 9—Italy where MU enhances collaboration of operators and end users determining better management, protection and sustainable use of MPAs). The same pattern exists for Social Added Values with the highest scores identified in case study 3 (Germany—leaving free space at sea by combining uses would benefit communities in the long run), case study 1 (North Scotland—MU promoting longevity of the fisheries sector, boosting innovation in the sector, increasing the trust of fishermen, and benefiting the local communities at large), and case study 5 (in the Algarve, Portugal—opportunities for business at family level, conservation of traditional activity and their culture and promotion of a healthy seafood diet).

### Analysis of MU by type of combination

**Fig 7:**
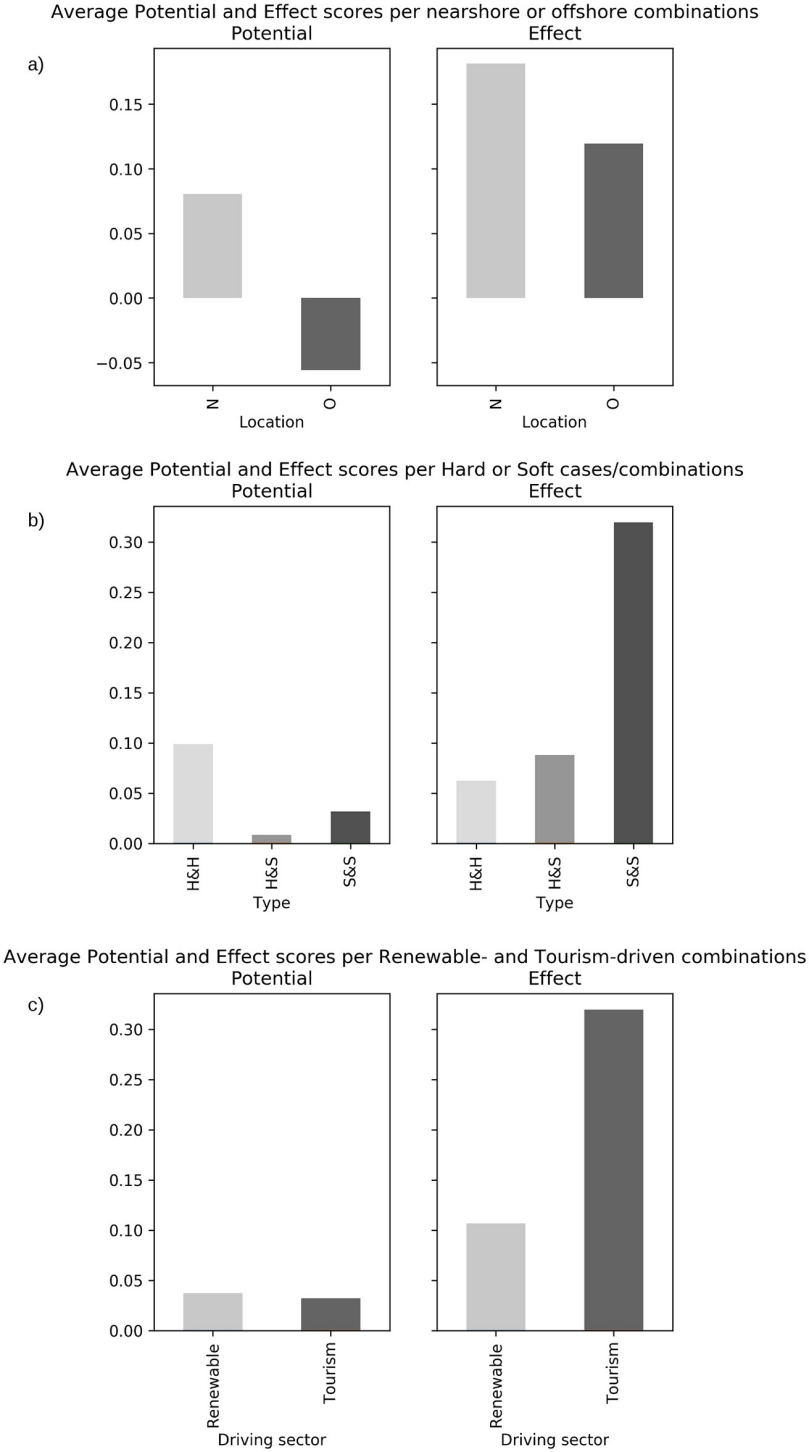
**Comparison of MU Potential and MU Effect between different characteristics of combinations:** (a) Nearshore vs Offshore, (b) Hard vs Soft, and (c) Renewables-driven vs Tourism-driven.

In order to provide a general understanding of what types of MU are seen as more promising across European Seas, the average MU Potential and MU Effect of different groups of combinations were compared (Fig 7) based on some of the MU characteristics identified in Table 4: MU location (Nearshore vs Offshore), MU type (Hard uses vs Soft uses), and MU driving sector (renewables-driven MU vs tourism-driven MU).

Considering the location of the MU (Fig 7a), both groups show MU Potential values close to 0. The average Potential for Nearshore combinations is slightly positive while the one for Offshore combinations is slightly negative, suggesting the offshore environment is more difficult to be exploited by two or more combined uses. Furthermore, this may also suggest that effective spatial management has an increased demand in nearshore environments where there is greater competition, and thus MU is viewed more positively closer to shore. Additionally, both show positive values of MU Effect, with Nearshore combinations associated to higher values of MU Effect.

With regards to the type of MU (Fig 7b), values are low for both Hard&Hard and Soft&Soft MU. Nevertheless, MU Potential of combinations considering two hard uses are approximately three times higher than the one involving two soft sectors. This can be explained by operators of hard sectors (e.g. renewable energy producers and large-scale aquaculture operators, utilising engineering infrastructures at sea) being considered by stakeholders more prepared than those of soft sectors (e.g. tourism and environmental protection) to face challenges involving combinations of different uses (including: technological changes, economic investments, administrative processes). The lowest MU Potential is obtained by combinations considering both hard and soft uses, where two very different approaches/traditions of the use of the sea have to be combined. It is worth noting that MU Effect results are dissimilar, with the highest values being attributed to the combination of two soft uses (about three times higher than for Hard&Hard). Similarly, combinations with both hard and soft uses obtained higher values of MU Effect than combinations with two hard uses.

Finally, the comparison of Renewables-driven with Tourism-driven combinations (Fig 7c) shows that both groups have positive values of MU Potential, with comparable values, and both show positive MU Effect scores, with the Tourism-driven group prevailing by a factor of about two.

### Key actions to be undertaken to promote MU development

Results from desk analysis and the stakeholder engagement processes in the case studies allowed for the identification of a set of recommended actions (S1 Table) to promote the implementation, or the strengthening, of MU. Recommendations are related to the aspects described among drivers or barriers in the DABI analysis. Therefore, this suggests practical ways to reinforce the factors promoting MU development and overcome those limiting it. The recommendations identified for the most relevant combinations are summarised in the following 10 themes.

**Policy, strategies, and planning**—10 of the 16 MU combinations referenced the need to promote and incorporate MU in the MSP process (MSP policy is identified among driver of MU). MU concepts can be promoted for example through identification of zones suitable for MU, by assigning preference towards MU versus single uses, or by ensuring appropriate representation of stakeholders. Marine renewable energy (MRE) combinations have placed greater emphasis on reduction targets for GHG emissions at the EU, national, and local levels in order to demonstrate the benefits of co-location and uptake for climate change agendas (renewable energy policy is identified among drivers of MU). Furthermore, the majority of MU combinations including tourism (4/7), located in the southern part of the Eastern Atlantic and the Mediterranean Sea, have referenced the need to create and/or improve regional sectoral policies focused on removing barriers to these MUs and targeting cross-sector needs and opportunities.

**Legal framework and administrative issues**—MU combinations which address these issues are those which are either driven by tourism or offshore wind energy. Barriers due to lack of, or restrictive, legislation and to the complexity of administrative procedures were identified for these combinations. The need for more consistent legal and administrative framework for MU at national and sub-national levels was identified, e.g the need to harmonize safety legislation for pescatourism (Azores, Italy) or regulation of different type of aquaculture (Denmark).

**Funding**—The implementation of a government subsidy in order to make MRE more competitive in the electricity generation market, thereby enhancing their commercial and economic viability, was indicated. Given the pre-commercial status of MRE technology in relation to other uses of the sea, these subsidies play an essential role in allowing for the implementation of MRE with any other use [29].

**Research and data production**—Offshore wind energy combinations emphasised the promotion of data-sharing protocols for health and safety considerations (e.g. fishing within offshore wind energy production areas), and to disseminate environmental baseline data, which may provide a greater knowledge base for environmental monitoring and protection, help in obtaining public understanding and acceptance, and provide knowledge transfer amongst technology and project developers [28]. Moreover, in-depth assessments of the cumulative economic, social and environmental impacts of different MU combinations (including the tourism-driven ones), and proof-of-concept and business models, are required in order to encourage financial investment.

**Technical improvements and innovation**—The tourism-driven combinations (T&F, T&A, T&E, T&H) recommend that the best type of boats for developing MU be identified, accomplishing requirements from commercial sectors and the need to host tourists on board (barriers to MU were identified in relation to the lack of suitable boats). Renewables combinations located in the northern part of the Eastern Atlantic and Baltic Sea target the need for the performance of particular innovative studies: on moorings, cable installation, fishing-friendly cable protection, connection of offshore energy to ports and on SSE, etc. For WI&F, equipment such as moorings, cable installation method, fishing-friendly cable protection measures and gear modifications should be further explored. For S&R, the connection of offshore energy to ports and on SSE generated from offshore renewables should also be further explored (technical barriers were identified as constituting a major obstacle for this combination).

**Pilot projects**—The need for promoting pilot projects and testing sites in order to enhance the general knowledge base and remove social barriers was identified, with offshore wind energy combinations in the North Sea specifically adding that small-scale pilot projects should be exempt from full-scale assessments.

**Networks and clusters**—It is suggested that a joint and cohesive approach to developing and strengthening MU is required at the EU, national, and local levels, whereby multi-sectoral and transdisciplinary actors can work together to overcome barriers and exploit mutual benefits.

**Dialogue and cooperation**—The need for dialogue and active cooperation resulted from the most diverse and numerous recommendations across cases and combinations. Tourism-driven combinations aim to facilitate dialogue between regulators/policy makers, academia and industry/commercial sectors, and amongst different sectors (e.g. to develop project ideas to pilot/implement MU through already available opportunities). Knowledge exchange on the success of MU practices was identified as a need, as well as integration and coordination both horizontally (between different sectors) and vertically (across governance levels, from EU to local). In the case of W&F in the North Sea, there is a specific reference to the need for a cross-border exchange with regulators of bordering countries where this combination exists already (i.e. UK/Denmark) in order to find commonalities and streamline management approaches.

**Education and training**—The most frequently cited recommendation from those case studies considering combinations with tourism was the promotion of education to sector operators. In addition, T&F, T&E, T&A also highlighted the need for training and capacity building for sector operators. MRE combinations located in the northern part of the Eastern Atlantic also demonstrated a theme of advocating for local stakeholder involvement in participatory planning and decision-making processes, which in turn would ideally resolve issues of local residents and communities objecting to MRE developments, potentially due to mistrust in decision-makers [41].

**Communication and social awareness**—The most commonly cited recommendation across cases and combinations was to promote the culture of the sea (including seamanship tradition, expertise, professions, historical marine routes, etc.), which occurred for all tourism-driven combinations (T&F, T&A, T&E, T&H). This recommendation is underpinned by the need to promote and market MU and its benefits, including the involvement of social media, to spread the MU concept, and to favour data access. The most prevalent recommendations for renewables-based MU combinations target the engagement of local stakeholders for effective dissemination of results and existing knowledge. This theme may be in line with Education and Training, whereby the involvement and empowerment of local communities in renewable energy development projects, particularly marine developments which suffer from greater unknowns concerning environmental, social, and economic impacts relative to onshore technologies and developments [42], is essential in order to secure community buy-in and facilitate project implementation [43].

The 10 case studies revealed differences in the typology of recommendations addressed, mostly due to the most promising MUs selected, but also to local and national requirements. For example, the need for establishing or reinforcing “network and clusters” of operators was pointed out only in case study 9 (Italy), where fragmentation of sectors involved in the most promising MUs (tourism, small scale fisheries and aquaculture, small and fragmented marine protected areas and underwater cultural heritage sites) poses a major barrier to the implementation of commercially viable MU initiatives. Even when significant commonalities emerged among the cases, the local and national context might have played a role in prioritising other specific issues in some of the case studies. This is an example of the recommendation typology “Dialogue and cooperation” which was pointed out by most of the case studies (seven out of ten). However, this typology was not highlighted in case study 1 (where national specific issues related to marine planning, marine licensing and funding were instead raised), case 6 (where the need for education and training of the sectors due to the limited educational level in the specific local context was strongly highlighted) and case 7 (where knowledge gaps linked to the implementation in the peculiar context of the Baltic sea, favourable legislative framework and need for pilot projects were mentioned).

## Discussion

### On the methods used and their influence on results

Some methodological limitations, mainly due to characteristics of data collected, should be acknowledged when comparing the results of different case studies. Heterogeneity of data types is an issue, as available data derived from different steps of the methodology are either qualitative (e.g. identification of DABI factors) or quantitative (scores attributed to DABI factors). Concerning the latter, scores represent an individual quantification of DABI factors, influenced by stakeholders’ opinion, knowledge and experience, so that a certain degree of subjectivity is included in the scoring process. However, subjectivity is partially offset by the fact that various stakeholders contributed to the identification and scoring of DABI factors related to the same MU combinations. In any case, this methodological limitation shall be taken into account in the evaluation of results, their integration and interpretation. Moreover, scoring methodology and the formulas adopted to estimate MU Potential and MU Effect could also be a possible source of uncertainty. For example, the average Driver and Barrier scores are not responsive to differences in the total number of drivers and/or barriers identified. If both DABI groups have a substantially different number of factors they would weigh the same regardless in the MU Potential calculation. In addition, heterogeneity across case studies should be considered, such as the number of interviews performed and number of factors included in the DABI catalogues. Furthermore, when aggregating results from case studies, for example by combination, the number of available data (e.g. scored DABI factors) may vary, since the various cases undertook different numbers of interviews.

Variability of scores across cases was considered by introducing the CV, used to provide an estimation of the degree of confidence associated to the values of MU Potential and MU Effect.

When considering the possible extension of the methodology to other contexts, some considerations should be take into account, which are also relevant for the interpretation of the results of the performed analysis. The overall low score for MU Potential is most likely linked to the low degree of implementation of MU in the case study areas we considered (and, at present, in EU seas in general [44]). Most cases considered potential MU combinations (see Table 5), with only some examples of implementation (mostly tourism-driven). However, the difference between potential and implemented MU combinations is limited, as in most of the cases the latter still needs to be widened and strengthened to reach the proper level of maturity required by successful commercial activities. This is confirmed by the average scores of MU Potential which are low and equal to 0.04 for both MU combinations categorised as implemented and as potential in Table 5. In addition, given that scores are assigned by stakeholder perceptions, there might be a general tendency to balance drivers and barriers of new opportunities. Instead, the predominantly positive score for MU Effect may have emerged by the general tendency of stakeholders to envisage the benefits of a new—and poorly known—approach to the use of the sea, instead of the negative impacts. Again, this may be related to the limited experience of MU implementation in EU seas (e.g. deployed pilots).

### MU combinations with more opportunity to be developed

Twelve out of fourteen scored MU combinations include either renewable energy or tourism as one of the sector involved in MU, while the other two involve both sectors. Renewable energy and tourism can be considered the two driving sectors for the advancement of the MU concept, with important geographic distinctions addressed in the next section. One of the aims of the study was to provide knowledge on which of these most promising MU combinations have a greater opportunity to be developed and/or strengthened. Such opportunity is defined by the MU Potential; when it is positive, drivers prevail on barriers, according to the stakeholders view, therefore providing a more favourable environment for stimulating the first and removing the second. The study also aimed to identify MU combinations which are seen as more beneficial from an environmental and socio-economic perspective (i.e. those with the higher MU Effect).

As demonstrated by the results of the analysis, the seven MU combinations that have received a positive MU Potential score (Fig 3), and subsequently those with the greatest opportunity for development or strengthening are driven by both offshore renewable energy (TI&M; R&D; TI&E; WI&F), and tourism (T&E&H; T&E; T&H). For renewables, it is noteworthy to mention that most (3 out of 4) of the combinations with positive MU Potential score were those which did not specifically focus on offshore wind energy as the only form of renewable energy being analysed. At the same time, all the three MU combinations with the lowest (negative) MU Potential (WI&E&T; WI&T; WI&A) include offshore wind energy as one of the sectors. The analysis of offshore wind energy-related MU combinations highlighted important barriers that, according to stakeholders’ perspective, limit MU Potential (Fig 3). Significant barriers affecting MU combinations involving offshore wind energy concern, for example, a lack of business case demonstrating the real advantages of MU involving offshore wind energy generation, additional financial costs for offshore wind energy developers (e.g. related to foundation types, installation methods, additional protection methods, cable routing, etc.), or high insurance costs for small-scale fisheries companies against possible damages to offshore wind installations. Conversely, the two tidal energy combinations obtained positive MU Potential scores, suggesting that this form of renewable energy production may be better suited to accommodate MU. However, it should be noted that MU assessment is more realistic in the case of offshore wind energy, as the sector has obtained a commercial status globally, while it is more speculative in the case of the relatively immature tidal energy sector. It is possible therefore that for less implemented forms of renewable energy, barriers were partially underestimated. Furthermore, offshore wind energy-related combinations were all located offshore (Table 5), where the highest scoring combinations, including tidal energy, were located nearshore, which suggests that the distance of renewables from shore might play a role in providing an opportunity for MU implementation. In fact, this is also evident when considering all the fourteen (renewable energy and tourism driven) combinations together, as shown in Fig 7a highlighting a MU Potential higher for Nearshore combinations.

The national and sub-national context can also play a role in creating a supporting environment for MU development. Three of the five MU combinations located off the coast of Scotland (TI&E and TI&M in case study 2, and WI&F in case study 1) were associated to a positive MU Potential score, while a fourth one (WA&A in case study 4) showed a balance between Drivers and Barriers (MU Potential = 0). The only Scottish MU combination with a negative MU Potential was S&R (case study 4). Indeed, interviewed stakeholders pointed out a high number of specific barriers for this MU combination; for example, technical barriers related to energy transmission and storage in ports or unsteady supply due to fluctuation of renewable energy production, as well as economic barriers linked to significant investments needed to convert vessels to shore side electricity or to diversify energy supply in ports strongly depending on non-renewable energy sources [31]. The relatively higher MU Potential of Scottish MU combinations might suggest that the convergence of a strong MSP process with marine renewable energy policies and legislation can act as a key factor towards the success of MU implementation, specifically concerning renewable energy.

Considering all the fourteen (renewable energy- and tourism-driven) combinations, those involving both hard and soft uses were associated to a lower MU Potential on average, while combinations with homogenous typologies of uses showed higher potential, with Hard&Hard MU having scored higher than Soft&Soft ones (Fig 7b). This pattern seems to play a role also when only renewable-energy driven combinations are considered. The two highest scored energy-driven combinations are related to renewables other than offshore wind energy when paired with other hard uses (TI&M; R&D). As discussed at the beginning of this section, offshore wind energy combinations performed worse than the above ones in terms of MU Potential, which in most case was also negative. Most of them (WI&F; WI&E&T; WI&A for case study 7 in the Baltic Sea—Sweden) were paired with soft uses, with the only exception of WI&A for case study 3 in the North Sea—German EEZ. This confirms MU combinations are more viable when co-located uses are of the same type (i.e. Hard & Hard, Soft & Soft). Regarding combinations with renewables, factors such as abiotic and/or biotic, growing and/or static, traditional and/or new, or having potential or being in-place (Table 5), do not seem to demonstrate a consistent relationship towards successful high MU Potential combinations.

**Fig 8:**
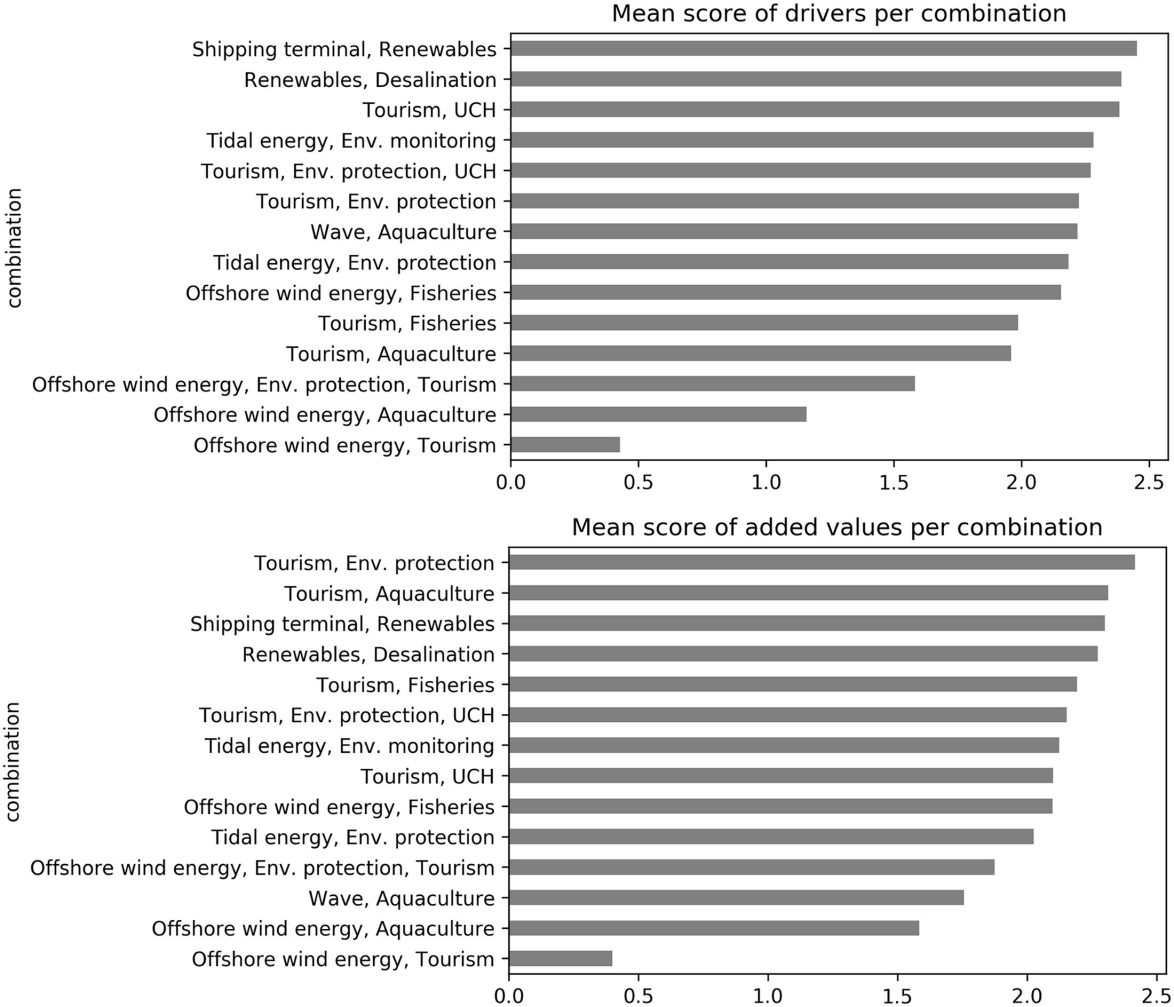
**Comparison of MU Combinations:** (Top) ranked by Drivers—Highest to lowest, and (Bottom) ranked by Added Values—Highest to lowest.

When viewing the opportunities for renewables-based MU combinations emanating from average driver scores (Fig 8—Top), as well as combinations which demonstrate the greatest benefits if implemented emanating from average added value scores (Fig 8—Bottom), many of the themes which are prevalent for MU Potential scores are further strengthened.

S&R, R&D and TI&M demonstrated the greatest driver scores for the group of renewable energy-driven combinations, and are also characterised by high added value scores. None of these combinations focus exclusively on offshore wind energy. On the contrary, coherently with the MU Potential, offshore wind energy combinations demonstrated lowest drivers and added values scores (in particular for WI&T), with the partial exception of WI&F. As mentioned previously, all offshore wind energy combinations are located offshore, whereby 2/3 of the highest scoring (for drivers and added values) renewable energy combinations (S&R; TI&M) are located nearshore and all three are paired with other hard uses, thus confirming the patterns discussed for MU Potential.

An analysis of the highest scored drivers for each of these three combinations do not demonstrate any clear thematic consistencies either for individual factors or categories. The most significant drivers for S&R, for example, include strategic/nodal location of ports as part of energy hub and connection with energy grid or availability of city council energy plans. In the case of the R&D examples of top-ranked drivers are a lack of freshwater in island communities and water stress during the high tourism season, while for TI&M significant drivers include the necessity to build upon knowledge gaps pertaining to environmental interactions in relation to tidal energy development or to assist Scotland in becoming a world leader in sustainable technological innovation. No synergistic conclusions can be drawn from this analysis towards renewables-based MU drivers in general, even when considering only the two combinations (S&R and TI&M) in question located within Scottish jurisdiction. Rather, the lack of commonalities amongst drivers—and added values as well—would suggest that each MU combination must be analysed specifically. Drivers and added values do not only depend on the renewable energy type and the other use this is paired with, but are—in most cases—also site-specific, being significantly influenced by local conditions.

For tourism-driven combinations, the combinations paired with underwater cultural heritage and environmental protection (T&E&H, T&E, T&H) were those with positive MU Potential scores. All of these paired uses are categorized as soft (and traditional) uses. The two tourism-paired combinations including offshore wind energy (categorized as hard), either on its own (WI&T) or in combination with other uses (WI&E&T) received the lowest MU Potential scores. This suggests that in the case of tourism driven combinations there is greater potential in facilitating MU with other soft uses, confirming that uses are more viably co-located when they are of the same type (i.e. Soft&Soft in the case of tourism driven MU combinations and Hard&Hard in the case of renewable energy driven ones), as also indicated by the results obtained when considering all the 14 most promising combinations together (Fig 7b). Uses belonging to the same typology tend to have similar approaches to the exploitation of the marine space and resources, share similar needs (in the case of soft uses, for example: less technological and engineering solutions and greater importance of simplification and homogenisation of administrative procedures) and involve business companies of similar (economic) size, which in the case of soft uses is in general small compared to hard ones. Although it has not been specifically investigated in the study, social acceptance might play a role in supporting combination of soft uses (relatively to combination of soft and hard uses), in particular when environmental protection and preservation of underwater cultural heritage are concerned. For tourism-driven MU, prevalence of Soft&Soft combinations might not be only determined by minor impacts on the marine environment and cultural heritage, but also by important added values which have been identified during the analysis, as for example co-management and co-monitoring of marine protected areas and underwater cultural heritage sites involving tourists and tourist operators (e.g. divers and diving centers) or integrative source of income for coastal communities, specifically in the case of operators of small-scale fisheries and mussel aquaculture (both considered as soft uses in the analysis).

The three tourism-related combinations with higher MU Potential scores (T&E&H, T&E, T&H) are also those in which tourism acts as the driver for the MU. Conversely combinations involving MU as a secondary sector, and which are driven by offshore wind energy, were characterised by the lowest MU Potential. This may suggest that tourism is more viable for MU implementation when the demand for touristic activities is particularly high and tourism is the primary use. As further discussed in the following section, this issue has also a geographical implication, as lower-scoring offshore wind energy and tourism combinations (WI&E&T; WI&T) are both located in the Baltic Sea, which is an area more vocated to renewables production, while higher-scoring combination of tourism with soft uses are located in the southern part of Eastern Atlantic and in the Mediterranean, where tourism is the greater economic driver. Considered cases studies mainly refer to staggered MU development, where one use is already in place and acts as main driver of the MU and another one is additionally integrated. Such form of MU development differs from joint MU development where two or more uses are together planned and developed as part of the same process [6]. Although we can expect the first being more feasible to be implemented, this aspect was not deeply evaluated in the present study and calls for additional research.

All tourism combinations with soft uses are located nearshore, whereas the comparatively lowest-scored tourism combinations with offshore wind energy are located offshore, which is in-line with the relationships suggested for renewable-driven combinations whereby the distance from shore of renewables acts as an indicator towards the difficulty of MU implementation.

When viewing the opportunities for tourism-based MU combinations resulting from average driver scores, most of the issues which have been pointed out in the discussion of the MU Potential are further strengthened, T&E&H, T&E, and T&H (Fig 8—Top) are the tourism-driven combinations which ranked first in terms of driver scores. An analysis of the highest scored most significant drivers for each of these three combinations, occurring in the southern part of Eastern Atlantic and the Mediterranean, confirms the importance of sustainable exploitation of the marine space (i.e. while ensuring protection to MPAs preservation of underwater cultural heritage) in order to further promote the economic benefits attainable by the expansion and diversification of touristic activities. National legislation and regulations concerning conservation management are also uniformly scored high across all case studies. In addition to the three combinations discussed above, T&A and T&F are among the tourism-driven combinations that were associated to higher added values scores, and which therefore would obtain the greater benefits if implemented. Along with the former combinations, T&A and T&F are also concentrated in the southern part of the Eastern Atlantic and in Mediterranean, with tourism being the driving sector for MU implementation, combined with other soft uses. An examination of the highest scored added values for each of these five combinations demonstrates several common themes. Nearly all combinations referenced economic gain, particularly at the local level, through both increased jobs and the diversification of tourism offer, as amongst the highest scored benefits of MU implementation. Most of the combinations also referenced one form of protection and conservation, whether it be environmental and/or cultural, accompanied by an increased awareness of sectoral cultural operations.

### Addressing MU development

The case study analysis pointed out that renewable energy and tourism are the two driving sectors for MU advancement in Europe in the near-future. It is therefore reasonable that MU is developed and strengthened around these uses, also considering their different geographic distribution reflecting their state of development in the various European sea basins. In this perspective, renewable energy-based combinations should be the focus of MU development in the North Sea, in the Baltic Sea and in the northern part of the Eastern Atlantic, whereas tourism-based combinations are more promising in the southern part of the Eastern Atlantic and in the Mediterranean Sea. In order to develop renewable energy-based combinations in the near-term, renewables infrastructure should be preferably paired with other hard uses, while tourism-based combinations should include other soft uses. Both renewable energy and tourism-driven combinations should probably focus near-term MU development on nearshore combinations, as offshore combinations are scored less favourably.

Future development of maritime sectors is highly dynamic, as also driven by various European initiatives on the Blue Economy (e.g. the Blue Growth Strategy [1]). This implies that multi-use of the sea space is a dynamic concept as well: if actively promoted, new combinations currently less feasible and socially acceptable could emerge as promising in the longer term, in line with the development of emerging maritime sectors. For the case of the Mediterranean Sea, these might include renewable energy-driven MU combinations (e.g. floating offshore wind energy & aquaculture, floating offshore wind energy and environmental protection, wave energy & aquaculture) as well as the re-use of decommissioned O&G infrastructure (specifically in Italy) in a MU perspective (e.g considering offshore wind energy, aquaculture and tourism) [45].

In order to facilitate MU development and strengthening in the longer term, a key element to target seems to be the amalgamation of the renewable energy and tourism industries. To facilitate synergies between the two, more research should be undertaken to determine the viability for compatibility of specific elements of these different sectors. As discussed in the previous section, the Scottish case studies seem to suggest that converging policy, legislative and planning processes can play a beneficial role for the development of renewable energy-driven combinations [29]. Countries which are still looking to develop the offshore renewable energy sectors, and have the potential to co-locate it with touristic activities could benefit from reviewing and tailoring the renewable energy-based legislative, policy and planning framework put in place by some of the northern European countries. Similarly, countries that have successfully adopted and implemented strong renewable energy plans and policies, and are looking to diversify their touristic offer, in order to inject revenues into local communities and subsequently enhance local job opportunities and economic stimulation, may benefit from reviewing and adopting the tourism-based plans and policies promoted by some of the countries which have more successfully implemented tourism offerings (i.e. those in the Mediterranean and in the southern part of the Eastern Atlantic). Finally, as the renewable energy industry further matures technologically, studies should be undertaken to evaluate and quantify the real versus the perceived risks to the health and safety of tourists when integrating tourism with renewable energy production, particularly concerning offshore location.

## Conclusions

The concept of MU of the sea has gained prominence in recent years due in-part to competing claims for space, initiating the investigation of suitable management policies and procedures for synergistic uses. This paper defined and applied a replicable methodology to analyse the characteristics and potential of MU development and strengthening through the implementation of quantitative and qualitative methods in site-specific local contexts. Despite the methodological limitations discussed, the applied methodology allowed to provide additional understanding about the RIs identified at the beginning of the study and is readily transferable to different European locations and marine management areas, supporting who are seeking to determine the state of knowledge of MU and target certain combinations for strengthening and development on micro and macro-economic scales.

Results from the aggregate analysis of contrasts and commonalities amongst MUs provide that renewable energy-driven and tourism-driven MU combinations demonstrated the greatest degree of potential implementation, with the former having prominence in the North Sea, Baltic Seas and northern part of Eastern Atlantic, and the latter in the Mediterranean Sea and the southern part of Eastern Atlantic. Generally, greater synergies are acknowledged between uses which are either both soft, as is the case of for tourism-driven MU, or hard, as is the case of energy-driven MU. Independently on the typology of the driving sector, the highest-scoring combinations in terms of MU Potential are located nearshore. Combinations including offshore wind energy generally received a lower MU Potential score, considering also that they have to be operated in an offshore marine environment, and are characterised by strong barriers related to construction, investment, risk, and health and safety issues. However, the analysis of drivers and added values suggested that each renewable energy type, and each use in which it is paired with, must be analysed specifically also considering the context of MU implementation. Whereby local site conditions may have great impact on successful implementation of the MU concept, as opposed to general policy-related, technical, environmental, or other regional and geographical considerations.

The most commonly cited recommendation across tourism-related cases and combinations was to promote the culture of the sea, including seamanship tradition, expertise, professions, knowledge of historical marine routes, etc. The most prevalent recommendation for renewable energy-based MU combinations target the engagement of local stakeholders for effective dissemination of results and existing knowledge and expected benefits.

Both recommendations provide insight into the need for education, training, communication, and social awareness amongst stakeholders with a vested interest.

Most analysed case studies identified that the existence of a legal, regulatory and planning framework supporting and enabling MU is a key component for real MU development and operation. However, the development and implementation of such frameworks, in Europe and beyond, will require clear guidelines and principles derived from the best available knowledge regarding MU. As shown by the case studies, the selection and development of the most promising MU combinations is very much dependent on local conditions. As MU develops in the future, policies and legislation can help frame societal drivers on EU, national, and local levels and eliminate barriers, while feasibility studies/pilot projects can help maximize synergies and mitigate negative impacts in order to allow MU to reach its optimum potential.

## Supporting information

S1 Table Recommendations from case studies for actions to promote MU development. (DOCX)
