# Supplementary material for: Multi-use of the sea: A wide array of opportunities from site-specific cases across Europe
Source: PLoS One. 2019 Apr 11;14(4):e0215010. doi: 10.1371/journal.pone.0215010 (PMC6459540; doi:10.1371/journal.pone.0215010)
Supplement: S1 Table — (DOCX) [file pone.0215010.s001.docx]

**Supporting information**

**S1 Table. Recommendations from case studies for actions to promote MU development. Numbers in brackets refer to case studies (see Fig.1 main manuscript).**

| **RECOMMENDATIONS** | **Tourism & Fisheries (case studies 5, 6, 9, 10)** | **Offshore wind & Aquaculture (case studies 3, 7, 8)** | **Tourism & Env. Prot. (case studies 5, 6, 9)** | **Offshore wind & Fisheries (case studies 1, C)** |
| --- | --- | --- | --- | --- |
| **Policy, strategies, planning** | To promote MSP the process (5), also at regional level (6)  To create and/or to improve regional sectoral policies focused on removing barriers to MU and targeting cross-sector needs and opportunities (9) | To address the MU concept and include it in MSP and local maritime and coastal development plans, assisting in the identification of areas suitable for establishing such MU combinations (8)   To create a national task force to determine the strategy and conditions surrounding the development of MU (8) | To promote the MSP process (5), also at regional level (6)  To create and/or to improve regional sectoral policies focused on removing barriers to MU and targeting cross-sector needs and opportunities (9) | To promote stronger coexistence policies in marine plans with explicit references to MU (1)  To encourage change from sectoral planning maps to "MU opportunity maps" (1) |
| **Legal framework & administrative issues** | To establish a general legal framework or a strategy for MU, facilitating licensing for joint activities or processes of risk assessment (5)  To create a more consistent legal and administrative framework focused on MU and its development (6)  To unify health care legislation between pesca-tourism and icthy-tourism at least at the regional scale (9) | To ensure a legislated claim for the secondary users in a MU scenario (3)   To clarify potential legislation and rules when combining different uses in marine areas (7) | To establish a general legal framework or a strategy for MU, facilitating licensing for joint activities or processes of risk assessment (5)  To create a more consistent legal and administrative framework focused on MU and its development (6)  To promote a change of attitude towards strategic and legislative instruments for marine ecosystems and biodiversity protection in order to exploit their potential as sustainable development opportunities (9) | To improve environmental assessment methodologies (1)  To require a co-existence plan and a mitigation strategy prior to the submission of a licence application (1)  To improve the current regulatory framework granting safety rights to fishermen (3) |
| **Funding** | To create targeted opportunities for developing MU in the framework of regional European funds, also including opportunities for acquiring suitable boats (9) | To ensure economic support for starting up and for maintaining pilot project activities long enough to gather valuable insights on the potential of MU (7) |  | To steer fundings towards MU applications and areas of technical innovation (1)   To encourage MU links between the offshore wind and the fishing industries within existing funding mechanisms (1) |
| **Research & data production** | To promote scientific research (5) | To promote research on the possibilities of cultivating mussels and algae in the Baltic and in combination with offshore wind power (7)  To encourage a transdisciplinary approach to gather information on MU as a topic (7)  To perform in-depth assessments of the impacts of the MU combinations and proof-of-concept and business models in order to encourage financial and investment interest (8) | To promote scientific research (5) | To perform empirical studies exploring the compatibility between offshore wind farms and commercial fisheries (1)  To promote data sharing agreements and protocols to demonstrate that fishing can take place safety within wind farms (1)  To fill research gaps for better mapping of navigational hazards, over-trawlability surveys (1) |
| **Technical improvements & innovation** | To identify the best type of boats for developing MU considering the meteorological and marine conditions in the area, and accomplishing requirements from commercial sectors (fishery, aquaculture) and the need to host tourists on board (9) |  | To identify the best type of boats for developing MU considering the meteorological and marine conditions in the area, and accomplishing requirements from commercial sectors (fishery, aquaculture) and the need to host tourists on board (9) | To perform innovation studies (e.g. moorings, cable installation method, fishing-friendly cable protection measures, gear modifications) (1)  To develop management strategies and technologies to minimise risks (3) |
| **Pilot projects** | To promote pilot projects and testing sites (5, 6) | To address the lack of a functioning full scale pilot facility to showcase the combination (3)  To promote pilot projects providing great knowledge resource (7) | To promote pilot projects and testing sites (5, 6) | To exempt small-scale pilot projects from full-scale assessments (1)  To follow the Scottish example of the “Survey, Deploy, and Monitor” (SDM) policy for ocean energy (1) |
| **Networks & clusters** | To create clusters of business operators to develop and implement MU, also including networks with local operators in the field of food supply (9) |  | To create a network of protected areas for coordinated management in relation to MU development (9) |  |
| **Dialogue & cooperation** | To enhance dialogue and create a mechanism for stakeholders to get together in order to participate in decision-making (5)To encourage inter-sectoral cooperation among different institutions, and among public institutions and economic operators to effectively manage new experiences of MU (9)To strengthen the interfaces between policy, science, industry and society in order to promote innovative concepts of MU (10)To strengthen horizontal and vertical integration of the different governance levels, through a truly participatory process (10)To disseminate successful MU practises and knowledge (5)To create joint working tables between institutions and commercial sectors to complete the analysis of MU opportunities in the area and identify the resources to be valorised through MU (9)To create working tables between commercial sectors to develop project ideas to pilot / implement MU through already available opportunities (9) | To facilitate clear and open communication between all involved stakeholders to promote the sharing of all available information (3)To encourage discussion about the potential in MU among academia, policy makers, local business and local NGO’s (7)To encourage cooperation among different sectors that are important for developing a MU (7)To favour cross sectoral multi-stakeholder dialogue (8)To further discuss the potential of the MU through meetings or workshop (7)To create physical opportunities for further discussion (8) | To enhance dialogue and create a mechanism for stakeholders to get together in order to participate in decision-making (5)To encourage inter-sectoral cooperation among different institutions, and among public institutions and economic operators to effectively manage new experiences of MU (9)To disseminate successful MU practises and knowledge (5)To create joint working tables between institutions and commercial sectors to complete the analysis of MU opportunities in the area and identify the resources to be valorised through MU (9)To create working tables between commercial sectors to develop project ideas to pilot / implement MU through already available opportunities (9) | Cross-border exchange with regulators of bordering countries where this combination exists already (i. e. UK, DK) to find commonalities and streamline management approaches.To encourage clear and open communication between both user groups and regulators (3) |
| **Education & training** | To provide training and capacity-building for MU (5)  To promote educational actions, e.g. foreign languages and entrepreneurship (6)  To create educational opportunities for business operators in fishing and aquaculture to train them for MU (9) |  | To provide training and capacity-building for MU (5)  To promote educational actions, e.g. foreign languages and entrepreneurship (6) | Further educational resources for commercial fishing to developers and contractors (1) |
| **Communication & social awareness** | To promote and market MU and its benefits, including the involvement of social media, to spread the MU concept, and to favour data access (6)  To promote the culture of the sea, including seamanship tradition, expertise, professions, historical marine routes, etc. (9)  To involve the local community in a truly transparent and participatory process, contributing to raising their awareness and the benefits of MU (10) | To engage local stakeholders for effective dissemination of results and existing knowledge (7) | To promote and market MU and its benefits, including the involvement of social media, to spread the MU concept, and to favour data access (6)  To promote the culture of the sea, including seamanship tradition, expertise, professions, historical marine routes, etc. (9) | To demonstrate the links of MU and Corporate Social Responsibility (1) |

| **RECOMMENDATIONS** | **Tourism & Aquaculture (case studies 5, 9)** | **Offshore wind & Tourism (case study 7)** | **Offshore wind & Env. Prot. & Tourism (case study 8)** | **Wave energy & Aquaculture (case study 4)** |
| --- | --- | --- | --- | --- |
| **Policy, strategies, planning** | To promote the MSP process (5)   To create and/or to improve regional sectoral policies focused on removing barriers to MU and targeting cross-sector needs and opportunities (9) |  | To address the MU concept and include it in MSP and local maritime and coastal development plans, assisting in the identification of areas suitable for establishing such MU combinations (8) | To adequately define MU, at EU, national and local levels, and provide explicit requirements and formal guidance for MU (4)   To link MUs to the objectives of GHG emissions reductions; To encourage EU, national and local level “leaders” to champion MU concept and deployment (4) |
| **Legal framework & administrative issues** | To establish a general legal framework or a strategy for MU, facilitating licensing for joint activities or processes of risk assessment (5)  To recognize aquaculture-related tourism as a business activity in regional legislation (9) | To clarify potential legislation and rules when combining different uses in marine areas (7) |  |  |
| **Funding** | To create targeted opportunities for developing MU in the framework of regional European funds, also including opportunities for acquiring suitable boats (9) | To ensure economic support for starting up and for maintaining pilot project activities long enough to gather valuable insights on the potential of MU (7) |  | To promote research funding authorities to avail adequate money for scaled-up development and deployment – to showcase commercial viability of MU (4)   To align MU development with insurance markets realities: the scope of an MU will differ from the scope of individual developers, especially relating to issues of health and safety and liability (4) |
| **Research & data production** | To promote scientific research (5) | To encourage a transdisciplinary approach to gather information on MU as a topic (7) | To perform in-depth assessments of the impacts of the MU combinations and proof-of-concept and business models in order to encourage financial and investment interest (8) |  |
| **Technical improvements & innovation** | To identify the best type of boats for developing MU considering the meteorological and marine conditions in the area, and accomplishing requirements from commercial sectors (fishery, aquaculture) and the need to host tourists on board (9) |  |  |  |
| **Pilot projects** | To promote pilot projects and testing sites (5, 6) | To promote pilot projects providing a great knowledge resource (7) |  |  |
| **Networks & clusters** | To create clusters of business operators to develop and implement MU, also including networks with local operators in the field of food supply (9) |  |  |  |
| **Dialogue & cooperation** | To enhance dialogue and create a mechanism for stakeholders to get together in order to participate in decision-making (5)  To encourage inter-sectoral cooperation among different institutions, and among public institutions and economic operators to effectively manage new experiences of MU (9)  To disseminate successful MU practises and knowledge (5)  To create joint working tables between institutions and commercial sectors to complete the analysis of MU opportunities in the area and identify the resources to be valorised through MU (9)  To create working tables between commercial sectors to develop project ideas to pilot / implement MU through already available opportunities (9) | To encourage cooperation among different sectors that are important for developing a MU (7)  To encourage discussion about the potential in MU among academia, policy makers, local business and local NGO’s (7)  To favour cross-sectoral multi-stakeholder dialogue (8)  To further discuss the potential of the MU through meetings or workshop (7) | To favour cross-sectoral multi-stakeholder dialogue (8)  To create physical opportunities for further discussion (8) | To maintain a dedicated on-line portal for MUs and EIAs in MUs, providing a significant mechanism for lessons learning and information exchange (4) |
| **Education & training** | To provide training and capacity-building for MU (5)  To create educational opportunities for business operators in fishing and aquaculture to train them for MU (9) |  |  | To coordinate a cross-sector group of actors to develop at a least one single scale-up showcase of success (4) |
| **Communication & social awareness** | To promote the culture of the sea, including seamanship tradition, expertise, professions, historical marine routes, etc. (9) | To engage local stakeholders for effective dissemination of results and existing knowledge (7) |  | To integrate local communities in MUs, resolving issues of local residents and communities objecting relevant developments (4) |

| **RECOMMENDATIONS** | **Tidal energy & Env. Prot. (case study 2)** | **Tidal energy & Env. Monitoring (case study 2)** | **Tourism & UCH (case study 9)** | **Tourism & UCH & Env. Prot. (case study 6)** |
| --- | --- | --- | --- | --- |
| **Policy, strategies, planning** | To develop policies and procedures informing how site-level trade-offs are to be made when siting tidal energy developments (2)  To restructure SEAs, EIAs, and MSP to consider synergies and negative impacts specific to MU with tidal energy, EPAs, and other uses/users of marine space (2)  To implement a standardized approval procedure whereby ENGOs agree on limits of potential impact and mitigation measures, and therefore eliminate potential litigation (2)   To restructure the licensing regime to allow for a streamlined process for staggered MU development between tidal energy projects and environmental protection, monitoring, and other uses (2) |  | To create and/or to improve regional sectoral policies focused on removing barriers to MU and targeting cross-sector needs and opportunities (9) | To promote MSP process also at the regional level for Azores (6) |
| **Legal framework & administrative issues** |  |  |  | To create a more consistent legal and administrative framework focused on MU and its development (6) |
| **Funding** | To replace the CFD mechanism with a subsidy which allows for tidal energy to be competitive with commercial offshore wind (2)  To provide subsidies for tidal energy developments in remote areas to access necessary grid infrastructure, provide for upgrades to existing infrastructure, and plan for the co-location of monitoring and electricity cable routes, thereby facilitating direct-to-shore connection of monitoring data (2)  To fund fisheries research in order to characterize fish movements, leading to enhanced TCT and fish management plans, individually and in relation to MU, through informed policy development (2) | To replace the CFD mechanism with a subsidy which allows for tidal energy to be competitive with commercial offshore wind (2)  To provide subsidization for improvements to monitoring equipment (2)  To provide subsidies for tidal energy developments in remote areas to access necessary grid infrastructure, provide for upgrades to existing infrastructure, and plan for the co-location of monitoring and electricity cable routes, thereby facilitating direct-to-shore connection of monitoring data (2)  To fund fisheries research in order to characterize fish movements, leading to enhanced TCT and fish management plans, individually and in relation to MU, through informed policy development (2) |  |  |
| **Research & data production** | To establish measures which standardize environmental monitoring data collection procedures (EU) (2)  To gather standardized baseline data for potential development sites prior to granting consents (2)  To establish a binding requirement for developers to disseminate environmental data as a condition of consent (2)  To determine the viability of TCT arrays acting as default no-fishing zones and artificial reefs, and how this may affect the environmental dynamics in the area (2) | To establish measures which standardize environmental monitoring data collection procedures (EU) (2)  To gather standardized baseline data for potential development sites prior to granting consents (2)  To establish a binding requirement for developers to disseminate environmental data as a condition of consent (2)  To determine the viability of TCT arrays acting as default no-fishing zones and artificial reefs, and how this may affect the environmental dynamics in the area (2) | To identify the actions needed to minimize interferences between maritime activities (including MU) and UCH (9)  To prepare a database of UCH for the Northern Adriatic Sea, identifying the sites suitable for regulated touristic use and the sites where access is to be prohibited (9) |  |
| **Technical improvements & innovation** |  |  | To identify the best type of boats for developing MU considering the meteorological and marine conditions in the area, and accomplishing requirements from commercial sectors (fishery, aquaculture) and the need to host tourists on board (9) |  |
| **Pilot projects** |  |  |  | To promote pilot projects to help remove social barriers (6) |
| **Networks & clusters** |  |  |  |  |
| **Dialogue & cooperation** |  |  | To encourage inter-sectoral cooperation among different institutions, and among public institutions and economic operators to effectively manage new experiences of MU (9)  To disseminate successful MU practices and knowledge (5)  To create joint working tables between institutions and commercial sectors to complete the analysis of MU opportunities in the area and identify the resources to be valorised through MU (9)  To create working tables between commercial sectors to develop project ideas to pilot / implement MU through already available opportunities (9) | To disseminate successful MU practices and knowledge (5) |
| **Education & training** |  |  |  | To promote educational actions, e.g. foreign languages and entrepreneurship (6) |
| **Communication & social awareness** | To fund research investigating the viability of economic benefits being captured within local/regional communities (2) |  | To promote the culture of the sea, including seamanship tradition, expertise, professions, historical marine routes, etc. (9) | To promote and market MU and its benefits, including the involvement of social media, to spread the MU concept, and to favour data access (6) |

| **RECOMMENDATIONS** | **Oil and gas and Tourism and Aquaculture (case study 9)** | **Oil and gas and Renewable energy (case study 9)** | **Renewable energy and Desalination (case study 1)** | **Shipping terminal and Green energy generation (case study 4)** |
| --- | --- | --- | --- | --- |
| **Policy, strategies, planning** | To develop a Master Plan, as a result of a joint effort of central and local administrations, operators of the sector, key local stakeholders and research institutions; to include the analysis of potential MUs in the framework of the on-going MSP process (9) | To develop a Master Plan, as a result of a joint effort of central and local administrations, operators of the sector, key local stakeholders and research institutions; to include the analysis of potential MUs in the framework of the on-going MSP process (9) | To adopt a clear strategic vision by national policy makers on key issues of concern related e.g. to the country's energy development agenda, enabling the implementation of policy agreements and facilitating suitable investments (10) | To adequately define MU, at EU, national and local levels, and provide explicit requirements and formal guidance for MU (4)   To link MUs to the objectives of GHG emissions reductions; To encourage EU, national and local level “leaders” to champion MU concept and deployment (4) |
| **Legal framework & administrative issues** |  |  |  |  |
| **Funding** | To get specific attention from national and EU research funding agencies to boost innovation and blue growth in the area (9) | To get specific attention from national and EU research funding agencies to boost innovation and blue growth in the area (9) |  | To promote research funding authorities to avail adequate money for scaled-up development and deployment – to showcase commercial viability of MU (4)  To align MU development with insurance markets realities: the scope of a MU will differ from the scope of individual developers, especially relating to issues of health and safety and liability (4) |
| **Research & data production** |  |  |  |  |
| **Technical improvements & innovation** |  |  |  | To ensure progress on the connection of offshore energy to ports and on shore-side electricity generated from offshore renewables (4) |
| **Pilot projects** |  |  |  |  |
| **Networks & clusters** | To create a joint effort of central and local administrations, operators of the sector, key local stakeholders and research institutions, in order to create the conditions for a MU development that goes beyond some episodic and small scale experiments (9) | To create a joint effort of central and local administrations, operators of the sector, key local stakeholders and research institutions, in order to create the conditions for a MU development that goes beyond some episodic and small scale experiments (9) | To strengthen the interfaces between policy, science, industry and society in order to promote innovative concepts of MU (10)  To strengthen horizontal and vertical integration of the different governance levels, through a truly participatory process (10) | To coordinate a cross-sector group of actors to develop at least one single scale-up showcase of success (4)  To establish a closer co-operation among port developers/harbour authorities and the competent authorities for the seabed for the offshore renewable energy component (4)  To maintain a dedicated on-line portal for MUs and EIAs in MUs, providing a significant mechanism for lessons learning and information exchange (4) |
| **Dialogue & cooperation** |  |  |  |  |
| **Education & training** |  |  | To involve the local community in a truly transparent and participatory process, contributing to raising their awareness and benefits of MU (10) | To integrate local communities in MUs, resolving issues of local residents and communities objecting relevant developments (4) |
| **Communication & social awareness** |  |  |  |  |
